# Associations of Social, Cultural, and Community Engagement With Health Care Utilization in the US Health and Retirement Study

**DOI:** 10.1001/jamanetworkopen.2023.6636

**Published:** 2023-04-04

**Authors:** Qian Gao, Jessica K. Bone, Feifei Bu, Elise Paul, Jill K. Sonke, Daisy Fancourt

**Affiliations:** 1 Research Department of Behavioural Science and Health, Institute of Epidemiology & Health Care, University College London, London, United Kingdom; 2 Center for Arts in Medicine, University of Florida, Gainesville

## Abstract

**Importance:**

There is growing evidence for the health benefits associated with social, cultural, and community engagement (SCCE), including for supporting healthy behaviors. However, health care utilization is an important health behavior that has not been investigated in association with SCCE.

**Objective:**

To examine the associations between SCCE and health care utilization.

**Design, Setting, and Participants:**

This population-based cohort study used data from the 2008 to 2016 waves of the Health and Retirement Study (HRS), a longitudinal panel study using a nationally representative sample of the US population aged 50 years and older. Participants were eligible if they reported SCCE and health care utilization in the relevant HRS waves. Data were analyzed from July to September 2022.

**Exposures:**

SCCE was measured with a 15-item Social Engagement scale (including community, cognitive, creative, or physical activities) at baseline (frequency) and longitudinally over 4 years (no, consistent, increased, or decreased engagement).

**Main Outcomes and Measures:**

Health care utilization was assessed in association with SCCE within 4 overarching categories: inpatient care (ie, hospital stays, hospital readmissions, length of hospital stays), outpatient care (ie, outpatient surgery, physician visits, number of physician visits), dental care (including dentures), and community health care (ie, home health care, nursing home stays, nights in a nursing home).

**Results:**

A total of 12 412 older adults (mean [SE] age, 65.0 [0.1] years; 6740 [54.3%] women) were included in short-term analyses with 2 years of follow-up. Independent of confounders, more SCCE was associated with shorter hospital stays (incidence rate ratio [IRR], 0.75; 95% CI, 0.58-0.98), greater odds of outpatient surgery (odds ratio [OR], 1.34; 95% CI, 1.12-1.60) and dental care (OR, 1.73; 95% CI, 1.46-2.05), and lower odds of home health care (OR, 0.75; 95% CI, 0.57-0.99) and nursing home stays (OR, 0.46; 95% CI, 0.29-0.71). Longitudinal analysis included 8635 older adults (mean [SE] age, 63.7 [0.1] years; 4784 [55.4%] women) with data on health care utilization 6 years after baseline. Compared with consistent SCCE, reduced SCCE or consistent nonparticipation in SCCE was associated with more inpatient care utilization, such as hospital stays (decreased SCCE: IRR, 1.29; 95% CI, 1.00-1.67; consistent nonparticipation: IRR, 1.32; 95% CI, 1.04-1.68) but lower levels of subsequent outpatient care, such as physician visits (decreased SCCE: OR, 0.68; 95% CI, 0.50-0.93; consistent nonparticipation: OR, 0.62; 95% CI, 0.46-0.82) and dental care utilization (decreased SCCE: OR, 0.68; 95% CI, 0.57-0.81; consistent nonparticipation: OR, 0.51; 95% CI, 0.44-0.60).

**Conclusions and Relevance:**

These findings suggest that more SCCE was associated with more dental and outpatient care utilization and reduced inpatient and community health care utilization. SCCE might be associated with shaping beneficial early and preventive health-seeking behaviors, facilitating health care decentralization and alleviating financial burden by optimizing health care utilization.

## Key Points

**Question:**

Is social, cultural, and community engagement (SCCE) associated with subsequent health care utilization among older adults in the US?

**Findings:**

In this cohort study of 12 412 US older adults, more frequent SCCE was associated with lower inpatient and community health care utilization but more interaction with outpatient and dental care in 2 years of follow-up. Longitudinally, compared with consistent engagement, individuals with decreased SCCE were more likely to use inpatient care but had fewer outpatient and dental care visits at 6 years of follow-up.

**Meaning:**

These findings suggest that SCCE may have a protective association in shaping beneficial early and preventive health care–seeking behavior and potentially facilitate the decentralization of the health care system, reducing demand for inpatient and community health care.

## Teaser

This cohort study assesses associations of social, cultural, and community engagement and health care utilization among older US adults.

## Introduction

Social, cultural, and community engagement (SCCE) refers to active participation in a broad range of activities, including arts, crafts, sports, volunteering, and social groups.^1^ This kind of engagement is a fundamental component of age-friendly environments.^2^ There is increasing evidence for the direct and indirect health benefits of SCCE across cultures, especially in older populations.^3,4^ For example, protective associations have been shown with the maintenance of both physical and mental health,^5,6^ cognitive function,^7^ health-related quality of life,^8^ life satisfaction,^9^ and longevity.^10^ A wide range of causal mechanisms has been proposed to explain these associations, including psychological, biological, social, and behavioral pathways.^11,12^ SCCE could thus modify profiles of health care utilization directly by alleviating the burden of morbidity.^13^ However, it also may work indirectly through the complex behavioral pathways of social relationships and the psychological and socioeconomic determinants of health care utilization.^14^

In relation to social relationships*,* SCCE can enhance social networks, social support, and social integration,^12^ all of which are associated with health care utilization via buffering stress, providing access to social resources (eg, informal advice and support), helping to connect individuals to professional services, and transmitting attitudes, values, and norms about help-seeking.^15,16,17^ However, the direction of these associations can go both ways, with potential for increased and decreased health care utilization, depending on the individuals and circumstances.^18^ In relation to psychological determinants, SCCE has been reported to offer health benefits and coping strategies for mental health service users^19^ and to facilitate the common recovery process by promoting positivity.^20^ Enhanced coping may support earlier, preventive use of health care, as well as facilitating other health behaviors that reduce the need for health care.

SCCE has been reported as associated with the management of mental illness^21^ and chronic diseases.^3^ SCCE also is associated with increased health literacy among older adults.^18^ In relation to socioeconomic determinants of health, socioeconomic disadvantage is associated, for many systemic and other reasons outside an individual’s control, with lower interaction with preventive health care.^22^ Although SCCE is itself patterned by socioeconomic circumstance,^23^ it is possible for people to experience equal health benefits regardless of their socioeconomic backgrounds.^6^ If SCCE can be designed for individuals from lower socioeconomic groups, it could potentially help to address inequalities in access to health care. In line with this, scaling up SCCE through social prescribing programs has been increasingly recognized as a potentially effective way of better serving marginalized or disadvantaged groups to facilitate their community engagement,^24^ addressing multiple social determinants of health and health needs holistically.^3^ Overall, the social and health benefits associated with SCCE thus may have cumulative and interactive associations with health care utilization.

However, despite extensive research on other social factors (including social isolation, networks, and support) and health care utilization,^25^ there has been only limited research into the associations of SCCE with health care utilization. A systematic review^26^ found mixed evidence on the associations between social prescribing programs referring patients to SCCE with health care utilization in the UK National Health Service setting. Several experimental studies have shown decreases in primary care visits and physician visits following patient engagement in SCCE or social prescribing of SCCE,^26,27^ as well as reductions in inpatient admissions, accident and emergency attendances, and secondary care referrals.^28,29^ However, other studies have not found evidence for associations, especially in primary care.^30^ Epidemiological evidence is also mixed. In a study by Brasher and Leedahl,^31^ individuals who engaged in leisure activities (eg, hobbies) and volunteering had lower home health care utilization but higher outpatient surgery and physician visits and no difference in hospital admissions. In a study by Burr and Lee,^32^ greater religious attendance (a form of SCCE) was associated with higher dental care utilization. However, these epidemiological results are limited by focusing on specific aspects of SCCE and cross-sectional data. It remains unclear whether health care utilization varies following engagement in SCCE. Further longitudinal investigations are needed to identify whether SCCE has the potential to address the unmet health needs of older populations, informing person-centered care and alleviating the burden of managing multiple health conditions. Consequently, this study aimed to examine the short-term (up to 2 years) and longitudinal (changes over 6 years) associations of SCCE with a wide range of inpatient, outpatient, dental care, and community health care utilization outcomes among a nationally representative sample of older adults in the US.

## Methods

This cohort study was approved by the University of Florida institutional review board and the University College London Research ethics committee. Informed consent was acquired from all participants. We followed the Strengthening the Reporting of Observational Studies in Epidemiology (STROBE) reporting guideline.

### Data Source and Sample

Data were drawn from the Health and Retirement Study (HRS), a nationally representative panel study of all community (noninstitutionalized) residents in the US aged 50 years and older, from 2008 to 2016.^33^ A rotating random 50% subsample of HRS participants were invited to complete the Leave Behind Psychosocial and Lifestyle Questionnaires (LBQ) every 2 waves (2008 and 2012 or 2010 and 2014). For our initial short-term analyses, we included all participants who responded to the SCCE questions in 2008 or 2010 and health care utilization in the following core wave (2010 or 2012), which provided a final sample of 12 412 participants. For subsequent longitudinal analyses, we included 8635 participants who responded to the SCCE questions in 2008 or 2010 and were also successfully followed up in both the next LBQ survey (2012 or 2014) and the following core wave (2014 or 2016). eFigure 1 in Supplement 1 provides details of sample selection.

### Measures

#### Exposures

The Social Engagement Scale captured engagement in a set of social, cultural, and community activities at baseline.^34^ We conducted an exploratory factor analysis of the 15-item scale in 2008 and 2010 (eTable 1 in Supplement 1). As indicated by this factor analysis, SCCE was operationalized by 4 subscales relating to engagement in community activities (ie, volunteering, charity work, educational courses, sports or social clubs, and meetings of nonreligious organizations), cognitive activities (ie, reading, word games, cards, and writing), home-based creative activities (ie, gardening, baking, cooking, crafts, and hobbies), and physical activities (ie, exercise and walking). Responses were recorded on a 3-point scale, including nonparticipation (0 points), monthly engagement (1 point), and weekly engagement (2 points). We calculated the mean level of engagement in activities for the overall scale and each subscale. Then we categorized changes in SCCE across waves (2008 and 2010 to 2012 and 2014) as consistent nonparticipation, consistent engagement, increased engagement, and decreased engagement.

#### Outcome

Health care utilization was measured every 2 years in the core questionnaire. As utilization was measured over the preceding 2 years, we used data from the core wave following each LBQ wave (2010 and 2012 or 2014 and 2016). We included 7 binary indicators of health care utilization in the last 2 years (yes or no), grouped within 4 overarching categories: inpatient care (ie, hospital stay and readmission to hospital), outpatient care (ie, outpatient surgery and physician visit), dental care (including dentures), and community health care (ie, home health care and nursing home stay). We additionally considered 3 count variables describing the amount of health care utilization, including inpatient care (length of hospital stay), outpatient care (number of physician visits), and community health care (nights in nursing home) in the last 2 years.

#### Covariates

We considered a range of potential predisposing, enabling, and needs confounders by adopting the Behavioral Model of Health Services Use by Andersen.^35^ The model is designed to understand and estimate the drivers of health care use and assess inequalities in accessibility. Predisposing and enabling factors include sociodemographic and psychological factors associated with health care use and the resources necessary for accessing health.^36^ We thus adjusted for age (years), gender (men or women), race and ethnicity (categorized as Black, White, and others [including American Indian, Alaskan Native, Asian or Pacific Islander, Hispanic, and other]), marital status (married or unmarried), life satisfaction (very, somewhat, or not satisfied), education (none, high school, college, or postgraduate), employment (employed, unemployed, or inactive or retired), and household wealth (quintiles). Race and ethnicity were self-reported and included in analysis because they are potential confounders in the associations between SCCE and health care utilization.

Needs confounders generally refer to individuals’ evaluated and perceived needs for health care, such as health-related factors.^36^ We adjusted for self-rated health status (good, fair, or poor), multimorbidity of self-reported diagnosed chronic illnesses (yes or no to having ≥2 illnesses, including diabetes, lung disease, cancer, heart conditions, high blood pressure, arthritis, complications from stroke, or others),^37,38^ difficulties with activities of daily living (yes or no),^37^ difficulties with instrumental activities of daily living (yes or no),^39^ and depression (yes or no; indicated by a score of ≥3 on the modified 8-item Centre for Epidemiologic Studies Depression Scale).^40^

### Statistical Analysis

We first examined the short-term associations of SCCE (2008 and 2010 waves) with different types of health care utilization over the following 2 years (2010 and 2012 waves). Then we estimated the longitudinal associations of changes in SCCE with different types of health care utilization at a 6-year follow-up in the 2014 and 2016 waves. For binary outcomes, logistic regression models testing the associations between SCCE and health care utilization are presented before and after adjustment for potential confounders. Predisposing and enabling factors were adjusted for first, followed by needs confounders. For count outcomes, which were overdispersed, the associations with SCCE were tested using negative binomial regression models.^41^ We first tested the associations of overall SCCE frequency with each of the outcomes and then explored which of the 4 elements of SCCE (community, cognitive, creative, and physical activities) were driving these associations. For participants missing data on covariates, we imputed data using multiple imputation by chained equations.^42^ We generated 10 imputed data sets (maximum missingness, 1.9%) (eTable 2 in Supplement 1). The imputation model included all variables used in analyses, and all covariates were successfully imputed. The 2008 and 2010 HRS cross-sectional LBQ weights were used, and analyses were conducted on weighted imputed data sets to minimize the impacts of nonresponse. Since the findings from imputed analyses did not differ to complete case analyses (eTables 3-6 in Supplement 1), results from imputed data are reported. Hypothesis tests were 2-sided, with *P* < .05 considered statistically significant for all analyses. All analyses were performed using Stata statistical software version 17.0 (StataCorp) and R Studio version 1.4.1103 (R Project for Statistical Computing). Data were analyzed from July to September 2022.

## Results

The short-term sample included 12 412 participants (mean [SE] age, 65.0 [0.1] years; 6740 [54.3%] women), with 1142 Black participants (9.2%), 10 587 White participants (85.4%), and 670 participants (5.4%) identified as another race or ethnicity. Overall, 3041 participants (24.5%) experienced a hospitalization, 1117 participants (9.0%) had been readmitted to a hospital after a discharge within the past 2 years, 11 419 participants (92.0%) had visited a physician, 2371 participants (19.1%) had undergone outpatient surgery, 8341 participants (67.2%) had used dental care, 918 participants (7.4%) had used home health care, and 360 participants (2.9%) had stayed in a nursing home in the last 2 years (Table 1).

**Table 1.** Baseline Characteristics of Study Populations

Characteristic	Participants, No. (%)^a^	
	Short-term (N = 12 412)	Longitudinal (n = 8635)
Age, y		
Mean (SE)	65.0 (0.1)	63.7 (0.1)
<60	4493 (36.2)	3359 (38.9)
60-69	4220 (34.0)	3169 (36.7)
70-79	2371 (19.1)	1546 (17.9)
≥80	1328 (10.7)	561 (6.5)
Gender		
Women	6740 (54.3)	4784 (55.4)
Men	5672 (45.7)	3851 (44.6)
Race and ethnicity		
Black	1142 (9.2)	734 (8.5)
White	10 587 (85.4)	7452 (86.3)
Other^b^	670 (5.4)	449 (5.2)
Marital status		
Married (including cohabiting)	7956 (64.1)	5794 (67.1)
Separated or divorced, widowed, or never married	4456 (35.9)	2841 (32.9)
Education		
None	1700 (13.7)	993 (11.5)
High school or GED	6640 (53.5)	4628 (53.6)
College	2644 (21.3)	1917 (22.2)
Postgraduate	1415 (11.4)	1097 (12.7)
Employment or occupation		
Employed	5275 (42.5)	4024 (46.6)
Unemployed or inactive	2420 (19.5)	1597 (18.5)
Retired	4717 (38.0)	3041 (34.9)
Household wealth, quintiles		
First (lowest)	2433 (19.6)	1684 (19.5)
Second	2532 (20.4)	1744 (20.2)
Third	2396 (19.3)	1684 (19.5)
Fourth	2495 (20.1)	1727 (20.0)
Fifth (highest)	2544 (20.5)	1787 (20.7)
Life satisfaction		
Very satisfied	8726 (70.3)	6269 (72.6)
Somewhat satisfied	3078 (24.8)	1995 (23.1)
Not satisfied	608 (4.9)	371 (4.3)
Self-rated health status		
Good	9470 (76.3)	6943 (80.4)
Fair	2160 (17.4)	1287 (14.9)
Poor	782 (6.3)	406 (4.7)
ADLs		
Dependent	1638 (13.2)	915 (10.6)
Independent	10 774 (86.8)	7720 (89.4)
IADLs		
Dependent	2160 (17.4)	1209 (14.0)
Independent	10 252 (82.6)	7426 (86.0)
Chronic illnesses		
Multimorbidity	6963 (56.1)	4559 (52.8)
No comorbidity	5449 (43.9)	4076 (47.2)
Depression		
Depression	2358 (19.0)	1468 (17.0)
No	10 054 (81.0)	7167 (83.0)
Social, cultural, and community engagement score, mean (SE)^c^		
Overall scale	0.76 (0.01)	0.78 (0.01)
Subscale activities		
Community	0.32 (0.01)	0.34 (0.01)
Cognitive	0.94 (0.01)	0.96 (0.01)
Creative	0.90 (0.01)	0.94 (0.01)
Physical	1.20 (0.01)	1.24 (0.01)
Health care utilization		
Hospital stay	3041 (24.5)	1857 (21.5)
Readmission to hospital	1117 (9.0)	587 (6.8)
Outpatient surgery	2371 (19.1)	1710 (19.8)
Physician visit	11 419 (92.0)	7979 (92.4)
Dental care	8341 (67.2)	6053 (70.1)
Home health care	918 (7.4)	458 (5.3)
Nursing home stay	360 (2.9)	147 (1.7)
Amount of health care utilization, mean (SE)		
Length of hospital stay, d	1.83 (0.07)	1.34 (0.07)
Physician visits, No.	9.58 (0.18)	8.81 (0.18)
Nights in nursing home, No.	2.75 (0.34)	0.59 (0.10)

Abbreviations: ADLs, activities of daily living; GED, general educational development; IADLs, instrumental activities of daily living.

^a^ Percentages are weighted based on imputed data sets.

^b^ Including American Indian, Alaskan Native, Asian or Pacific Islander, Hispanic, and other.

^c^ Range, 0 to 2 points; higher score indicates more frequent participation. Community activities included volunteering, charity work, educational courses, sports or social clubs, and meetings of nonreligious organizations. Cognitive activities included reading, word games, cards, and writing. Home-based creative activities included gardening, baking, cooking, crafts, and hobbies. Physical activities included exercise and walking.

### Short-term Associations

#### Inpatient Care

In fully adjusted models, over the 2 years following baseline assessment, more overall SCCE was associated with shorter lengths of hospital stays (incidence rate ratio [IRR], 0.75; 95% CI, 0.58-0.98), although the associations for overall admission and readmission to hospital were attenuated after adjusting for health-related confounders (Table 2). Examining the 4 types of SCCE separately, associations were the most consistent for greater creative and physical engagement, which were associated with reduced use of all types of inpatient care, including admission and readmission to hospital, even in fully adjusted models (Table 3).

**Table 2.** Associations of Overall Social, Cultural, and Community Engagement With Subsequent Health Care Utilization at Baseline

Model	Estimate (95% CI) (N = 12 412)^a^									
	Inpatient care			Outpatient care			Dental care and dentures, OR	Community health care		
	Hospital stay, OR	Length of hospital stay, IRR	Readmission to hospital (2-y), OR^b^	Outpatient surgery, OR	Physician visits, OR	No. of physician visits, IRR		Home health care, OR	Nursing home stays, OR	Nights in nursing home, IRR
Crude model	0.48 (0.41-0.56)	0.42 (0.33-0.53)	0.31 (0.25-0.39)	1.30 (1.11-1.52)	1.96 (1.51-2.54)	0.92 (0.82-1.04)	4.57 (3.91-5.34)	0.30 (0.23-0.40)	0.16 (0.11-0.25)	0.18 (0.08-0.40)
Model 1^c^	0.67 (0.57-0.78)	0.59 (0.46-0.76)	0.51 (0.41-0.65)	1.20 (1.01-1.42)	0.99 (0.76-1.29)	0.93 (0.83-1.05)	1.81 (1.54-2.13)	0.48 (0.36-0.62)	0.27 (0.17-0.41)	0.38 (0.18-0.78)
Model 2^d^	0.90 (0.76-1.07)	0.75 (0.58-0.98)	0.81 (0.64-1.03)	1.34 (1.12-1.60)	1.26 (0.96-1.66)	1.08 (0.96-1.21)	1.73 (1.46-2.05)	0.75 (0.57-0.99)	0.46 (0.29-0.71)	0.47 (0.22-1.01)

Abbreviations: IRR, incidence rate ratio; OR, odds ratio.

^a^ All analyses were based on weighted and imputed data sets.

^b^ During 2 years of follow-up.

^c^ Model 1 adjusted for predisposing and enabling factors (ie, age, gender, educational level, race and ethnicity, marital status, employment or occupation, household wealth, and life satisfaction).

^d^ Model 2 adjusted for confounders in model 1 and health-related covariates (ie, depression, multimorbidity, activities of daily living, instrumental activities of daily living, and self-rated health status).

**Table 3.** Associations of Specific Subtypes of Social, Cultural, and Community Engagement With Subsequent Health Care Utilization at Baseline

Model	Estimate (95% CI) (N = 12 412)^a^									
	Inpatient care			Outpatient care			Dental care and dentures, OR	Community health care		
	Hospital stays, OR	Length of hospital stay, IRR	Readmission to hospital (2-y), OR	Outpatient surgery, OR	Physician visits, OR	No. of physician visits, IRR		Home health care, OR	Nursing home stays, OR	Nights in nursing home, IRR
**Community activities** ^b^										
Crude model	0.70 (0.61-0.80)	0.76 (0.62-0.92)	0.64 (0.51-0.80)	1.10 (0.96-1.27)	1.55 (1.21-1.99)	1.07 (0.96-1.18)	2.71 (2.34-3.15)	0.67 (0.52-0.86)	0.42 (0.28-0.61)	0.41 (0.21-0.83)
Model 1^c^	0.87 (0.76-1.01)	0.97 (0.78-1.21)	0.89 (0.72-1.10)	1.04 (0.90-1.21)	1.05 (0.83-1.33)	1.11 (1.01-1.23)	1.50 (1.29-1.73)	0.90 (0.71-1.14)	0.60 (0.41-0.86)	0.71 (0.34-1.46)
Model 2^d^	0.98 (0.85-1.13)	1.07 (0.86-1.33)	1.07 (0.87-1.32)	1.08 (0.93-1.25)	1.15 (0.91-1.46)	1.17 (1.06-1.28)	1.46 (1.26-1.69)	1.07 (0.85-1.35)	0.75 (0.52-1.08)	0.71 (0.34-1.48)
**Cognitive activities** ^e^										
Crude model	0.93 (0.84-1.02)	0.88 (0.73-1.06)	0.85 (0.73-0.99)	1.22 (1.09-1.36)	1.67 (1.37-2.02)	1.13 (1.03-1.24)	1.67 (1.52-1.85)	0.81 (0.68-0.95)	0.90 (0.69-1.18)	0.71 (0.47-1.08)
Model 1^c^	0.98 (0.88-1.09)	0.93 (0.78-1.09)	0.95 (0.81-1.11)	1.14 (1.01-1.28)	1.09 (0.90-1.32)	1.07 (1.00-1.16)	1.11 (1.00-1.24)	0.81 (0.68-0.96)	0.87 (0.66-1.14)	1.35 (0.79-2.28)
Model 2^d^	1.08 (0.96-1.20)	0.96 (0.81-1.14)	1.11 (0.95-1.31)	1.17 (1.04-1.32)	1.15 (0.95-1.40)	1.10 (1.01-1.19)	1.09 (0.98-1.22)	0.95 (0.80-1.13)	1.09 (0.83-1.43)	1.93 (1.18-3.16)
**Creative activities** ^f^										
Crude model	0.65 (0.59-0.72)	0.57 (0.49-0.66)	0.50 (0.43-0.58)	1.17 (1.05-1.29)	1.20 (1.03-1.40)	0.90 (0.84-0.98)	1.79 (1.64-1.96)	0.46 (0.39-0.54)	0.24 (0.18-0.32)	0.28 (0.17-0.44)
Model 1^c^	0.80 (0.72-0.88)	0.76 (0.65-0.87)	0.68 (0.59-0.78)	1.14 (1.02-1.27)	0.90 (0.76-1.06)	0.91 (0.84-0.98)	1.17 (1.05-1.30)	0.65 (0.55-0.76)	0.38 (0.29-0.50)	0.26 (0.17-0.40)
Model 2^d^	0.92 (0.83-1.02)	0.87 (0.74-0.99)	0.84 (0.72-0.97)	1.19 (1.07-1.33)	1.00 (0.84-1.19)	0.97 (0.90-1.05)	1.13 (1.02-1.26)	0.81 (0.69-0.96)	0.49 (0.38-0.65)	0.34 (0.22-0.54)
**Physical activities** ^g^										
Crude model	0.70 (0.66-0.75)	0.61 (0.55-0.68)	0.56 (0.52-0.62)	1.01 (0.94-1.08)	1.13 (1.02-1.24)	0.85 (0.81-0.89)	1.76 (1.66-1.87)	0.59 (0.54-0.65)	0.52 (0.44-0.61)	0.48 (0.35-0.65)
Model 1	0.80 (0.75-0.85)	0.70 (0.63-0.77)	0.67 (0.61-0.73)	1.00 (0.92-1.07)	0.98 (0.88-1.09)	0.88 (0.83-0.92)	1.39 (1.30-1.48)	0.71 (0.64-0.79)	0.65 (0.55-0.77)	0.71 (0.52-0.98)
Model 2	0.91 (0.85-0.97)	0.78 (0.71-0.87)	0.79 (0.72-0.88)	1.05 (0.97-1.13)	1.09 (0.98-1.23)	0.95 (0.91-1.00)	1.36 (1.27-1.46)	0.84 (0.75-0.93)	0.77 (0.65-0.92)	0.66 (0.47-0.93)

Abbreviations: IRR, incidence rate ratio; OR, odds ratio.

^a^ All analyses were based on weighted and imputed data sets.

^b^ Community activities included volunteering, charity work, educational courses, sports or social clubs, and meetings of nonreligious organizations.

^c^ Model 1 adjusted for predisposing and enabling factors (ie, age, gender, educational level, race and ethnicity, marital status, employment or occupation, household wealth, and life satisfaction).

^d^ Model 2 adjusted for confounders in model 1 and health-related covariates (ie, depression, multimorbidity, activities of daily living, instrumental activities of daily living, and self-rated health status).

^e^ Cognitive activities included reading, word games, cards, and writing.

^f^ Home-based creative activities included gardening, baking, cooking, crafts, and hobbies.

^g^ Physical activities included exercise and walking.

#### Outpatient Care

Inversely, more frequent overall SCCE was associated with higher odds of outpatient surgery (odds ratio [OR], 1.34; 95% CI, 1.12-1.60), but the association with physician visits was attenuated after accounting for sociodemographic confounders (Table 2). When exploring specific types of SCCE, participants with more cognitive and creative activity engagement had higher odds of outpatient surgery utilization. Community and cognitive activity participation were also associated with an increased number of physician visits (Table 3).

#### Dental Care

Similarly, greater SSCE was associated with increased odds of dental care (OR, 1.73; 95% CI, 1.46-2.05) (Table 2). This finding was consistent for all types of SCCE except cognitive activity (Table 3).

#### Community Health Care

Participants with more SCCE had decreased odds of home health care utilization (OR, 0.75; 95% CI, 0.57-0.99) and nursing home stays (OR, 0.46; 95% CI, 0.29-0.71) (Table 2). This association was primarily observed for creative and physical activity engagement, which were also associated with fewer nights spent in nursing homes, although more frequent cognitive activities were associated with longer nursing home stays (Table 3).

### Longitudinal Associations

Longitudinal analyses included a total of 8635 participants (mean [SE] age 63.7 [0.1] years; 4784 [55.4%] women), including 734 Black adults (8.5%) and 7452 White adults (86.3%), and 449 participants (5.2%) identified as another race or ethnicity (Table 1). Spaghetti plots for mean levels of SCCE over follow-up are presented in eFigure 2 and eFigure 3 in Supplement 1.

#### Inpatient Care

Compared with consistent engagement, decreased engagement and nonparticipation in SCCE over the 6-year follow-up were associated with more inpatient care utilization; specifically longer hospital stays (decreased engagement: IRR, 1.29; 95% CI, 1.00-1.67; consistent nonparticipation: IRR, 1.32; 95% CI, 1.04-1.68) (Table 4). These associations were primarily driven by decreased and nonparticipation in physical activity (Table 5).

**Table 4.** Associations Between Changes in Overall Social, Cultural, and Community Engagement and Subsequent Health Care Utilization

Model	Estimate (95% CI) (n = 8635)^a^									
	Inpatient care			Outpatient care			Dental care, OR	Community health care		
	Hospital stay, OR	Length of hospital stay, IRR	Readmission to hospital, OR	Outpatient surgery, OR	Physician visit, OR	No. of physician visits, IRR		Home health care, OR	Nursing home stay, OR	Nights in nursing home, IRR
**Crude model**										
Increased engagement	1.27 (1.03-1.55)	1.50 (1.08-2.08)	1.24 (0.90-1.71)	0.93 (0.75-1.15)	0.63 (0.44-0.90)	0.93 (0.81-1.05)	0.50 (0.41-0.60)	1.12 (0.81-1.54)	0.95 (0.59-1.53)	1.29 (0.56-2.97)
Decreased engagement	1.34 (1.13-1.57)	1.97 (1.49-2.59)	1.66 (1.29-2.13)	0.97 (0.81-1.15)	0.60 (0.45-0.80)	1.06 (0.96-1.18)	0.47 (0.40-0.55)	1.54 (1.20-1.98)	1.78 (1.28-2.48)	3.47 (1.88-6.39)
Consistent nonparticipation	1.50 (1.30-1.73)	2.10 (1.71-2.57)	2.05 (1.65-2.54)	0.78 (0.67-0.91)	0.47 (0.36-0.61)	1.00 (0.90-1.12)	0.27 (0.23-0.31)	1.93 (1.55-2.39)	1.78 (1.32-2.40)	3.10 (1.73-5.56)
**Model 1** ^b^										
Increased engagement	1.16 (0.94-1.43)	1.19 (0.85-1.66)	1.08 (0.78-1.50)	0.97 (0.78-1.20)	0.77 (0.54-1.11)	0.92 (0.82-1.03)	0.69 (0.56-0.85)	1.05 (0.76-1.45)	0.87 (0.52-1.46)	0.77 (0.35-1.70)
Decreased engagement	1.13 (0.96-1.34)	1.48 (1.14-1.92)	1.37 (1.06-1.77)	1.00 (0.84-1.19)	0.73 (0.54-0.99)	1.07 (0.97-1.18)	0.66 (0.55-0.78)	1.26 (0.97-1.63)	1.29 (0.92-1.80)	1.70 (0.95-3.07)
Consistent nonparticipation	1.17 (1.00-1.36)	1.55 (1.23-1.95)	1.48 (1.18-1.86)	0.83 (0.70-0.98)	0.70 (0.53-0.93)	1.03 (0.93-1.14)	0.49 (0.42-0.57)	1.41 (1.11-1.78)	1.15 (0.83-1.58)	1.63 (0.89-2.99)
**Model 2** ^c^										
Increased engagement	1.12 (0.90-1.38)	1.15 (0.85-1.56)	1.01 (0.73-1.40)	0.94 (0.76-1.17)	0.74 (0.52-1.06)	0.90 (0.81-1.01)	0.70 (0.57-0.86)	0.98 (0.71-1.37)	0.83 (0.49-1.41)	0.94 (0.39-2.24)
Decreased engagement	1.03 (0.87-1.22)	1.29 (1.00-1.67)	1.20 (0.93-1.55)	0.97 (0.81-1.15)	0.68 (0.50-0.93)	0.99 (0.90-1.09)	0.68 (0.57-0.81)	1.10 (0.84-1.44)	1.14 (0.81-1.61)	1.49 (0.78-2.83)
Consistent nonparticipation	1.01 (0.87-1.19)	1.32 (1.04-1.68)	1.24 (0.98-1.56)	0.78 (0.66-0.93)	0.62 (0.46-0.82)	0.91 (0.83-0.99)	0.51 (0.44-0.60)	1.17 (0.92-1.50)	0.97 (0.69-1.37)	1.65 (0.86-3.14)

Abbreviations: IRR, incidence rate ratio; OR, odds ratio.

^a^ All analyses were based on weighted and imputed data sets. The consistent engagement group was the referenced group.

^b^ Model 1 adjusted for predisposing and enabling factors (ie, age, gender, educational level, race and ethnicity, marital status, employment or occupation, household wealth, and life satisfaction).

^c^ Model 2 adjusted for confounders in model 1 and health-related covariates (ie, depression, multimorbidity, activities of daily living, instrumental activities of daily living, and self-rated health status).

**Table 5.** Associations Between Changes in Specific Subtypes of Social, Cultural, and Community Engagement and Subsequent Health Care Utilization

Model	Estimate (95% CI) (n = 8635)^a^									
	Inpatient care			Outpatient care			Dental care, OR	Community health care		
	Hospital stay, OR	Length of hospital stay, IRR	Readmission to hospital, OR	Outpatient surgery, OR	Physician visit, OR	No. of physician visits, IRR		Home health care, OR	Nursing home stay, OR	Nights in nursing home, IRR
**Community activities** ^b^										
**Crude model**										
Increased engagement	1.23 (1.01-1.50)	1.37 (0.96-1.96)	1.05 (0.76-1.44)	0.92 (0.75-1.14)	0.76 (0.54-1.07)	0.90 (0.81-1.00)	0.53 (0.44-0.64)	1.25 (0.92-1.69)	0.67 (0.42-1.07)	0.57 (0.23-1.41)
Decreased engagement	1.24 (1.05-1.47)	1.44 (1.10-1.99)	1.46 (1.13-1.88)	0.89 (0.73-1.07)	0.76 (0.56-1.02)	1.01 (0.91-1.13)	0.52 (0.44-0.61)	1.31 (1.02-1.70)	1.54 (1.11-2.13)	2.46 (1.35-4.48)
Consistent nonparticipation	1.35 (1.17-1.55)	1.64 (1.33-2.01)	1.74 (1.42-2.14)	0.77 (0.66-0.90)	0.51 (0.40-0.64)	0.96 (0.86-1.07)	0.31 (0.28-0.36)	1.71 (1.39-2.10)	1.33 (1.00-1.77)	1.82 (1.00-3.31)
**Model 1** ^c^										
Increased engagement	1.14 (0.93-1.39)	1.16 (0.83-1.62)	0.91 (0.66-1.26)	0.97 (0.78-1.19)	0.96 (0.68-1.35)	0.90 (0.81-1.00)	0.74 (0.61-0.90)	1.18 (0.86-1.61)	0.60 (0.36-1.00)	0.34 (0.16-0.68)
Decreased engagement	1.07 (0.90-1.28)	1.29 (0.99-1.68)	1.24 (0.95-1.60)	0.92 (0.76-1.12)	0.99 (0.72-1.36)	1.04 (0.94-1.16)	0.74 (0.62-0.88)	1.10 (0.84-1.43)	1.17 (0.83-1.63)	1.45 (0.80-2.65)
Consistent nonparticipation	1.07 (0.92-1.24)	1.36 (1.09-1.69)	1.30 (1.04-1.61)	0.82 (0.69-0.97)	0.77 (0.59-0.99)	0.99 (0.89-1.11)	0.55 (0.48-0.64)	1.30 (1.03-1.63)	0.89 (0.65-1.22)	1.54 (0.84-2.84)
**Model 2** ^d^										
Increased engagement	1.07 (0.87-1.32)	1.09 (0.79-1.48)	0.84 (0.61-1.17)	0.95 (0.76-1.17)	0.92 (0.65-1.30)	0.87 (0.79-0.96)	0.76 (0.62-0.93)	1.09 (0.79-1.50)	0.54 (0.32-0.92)	0.28 (0.14-0.59)
Decreased engagement	1.00 (0.84-1.20)	1.16 (0.89-1.50)	1.12 (0.86-1.46)	0.91 (0.75-1.10)	0.94 (0.69-1.30)	0.99 (0.90-1.10)	0.76 (0.64-0.91)	0.99 (0.76-1.30)	1.04 (0.73-1.48)	1.31 (0.67-2.56)
Consistent nonparticipation	0.96 (0.82-1.12)	1.22 (0.96-1.54)	1.13 (0.91-1.42)	0.78 (0.66-0.93)	0.70 (0.54-0.91)	0.90 (0.82-0.98)	0.58 (0.50-0.67)	1.14 (0.90-1.44)	0.78 (0.56-1.09)	1.37 (0.72-2.63)
**Cognitive activities** ^e^										
Crude model										
Increased engagement	1.33 (0.83-2.12)	1.13 (0.60-2.14)	1.53 (0.79-2.93)	0.62 (0.32-1.20)	0.31 (0.17-0.56)	0.81 (0.59-1.11)	0.32 (0.21-0.49)	1.20 (0.59-2.44)	1.01 (0.35-2.90)	0.12 (0.04-0.42)
Decreased engagement	0.90 (0.60-1.35)	1.31 (0.81-2.14)	1.54 (0.92-2.56)	1.05 (0.66-1.65)	0.51 (0.32-0.79)	1.65 (0.97-2.80)	0.37 (0.26-0.52)	1.84 (1.13-2.97)	1.52 (0.75-3.08)	2.35 (0.66-8.39)
Consistent nonparticipation	1.51 (0.91-2.51)	1.67 (1.01-2.77)	1.94 (1.00-3.75)	0.97 (0.53-1.79)	0.35 (0.19-0.64)	1.02 (0.70-1.48)	0.30 (0.18-0.48)	2.71 (1.47-5.00)	1.21 (0.43-3.42)	2.81 (0.49-15.95)
Model 1^c^										
Increased engagement	1.14 (0.70-1.85)	0.79 (0.47-1.34)	1.18 (0.60-2.34)	0.71 (0.36-1.39)	0.55 (0.30-0.99)	0.81 (0.60-1.08)	0.66 (0.40-1.09)	0.99 (0.46-2.11)	0.98 (0.29-3.25)	0.61 (0.16-2.35)
Decreased engagement	0.74 (0.49-1.13)	1.19 (0.68-2.08)	1.18 (0.70-1.97)	1.21 (0.77-1.91)	0.88 (0.56-1.38)	1.82 (1.06-3.12)	0.78 (0.55-1.10)	1.50 (0.88-2.54)	1.22 (0.59-2.51)	0.63 (0.17-2.33)
Consistent nonparticipation	1.06 (0.64-1.78)	0.93 (0.53-1.63)	1.19 (0.61-2.33)	1.29 (0.68-2.43)	0.86 (0.43-1.71)	1.14 (0.76-1.69)	0.88 (0.50-1.53)	1.71 (0.88-3.32)	0.68 (0.24-1.89)	0.07 (0.01-0.39)
Model 2^d^										
Increased engagement	1.07 (0.64-1.79)	0.82 (0.50-1.35)	1.06 (0.52-2.18)	0.68 (0.34-1.36)	0.54 (0.29-0.99)	0.74 (0.56-0.97)	0.67 (0.41-1.11)	0.87 (0.39-1.94)	0.85 (0.24-2.94)	1.34 (0.29-6.19)
Decreased engagement	0.62 (0.40-0.95)	1.00 (0.56-1.77)	0.93 (0.55-1.58)	1.12 (0.72-1.74)	0.78 (0.49-1.24)	1.35 (0.95-1.92)	0.84 (0.59-1.19)	1.23 (0.72-2.10)	1.04 (0.50-2.17)	0.50 (0.13-1.96)
Consistent nonparticipation	0.85 (0.49-1.46)	1.11 (0.56-2.20)	0.87 (0.42-1.83)	1.16 (0.60-2.24)	0.72 (0.36-1.41)	0.95 (0.64-1.40)	0.99 (0.56-1.76)	1.22 (0.60-2.50)	0.46 (0.17-1.25)	0.04 (0.01-0.25)
**Creative activities** ^f^										
Crude model										
Increased engagement	1.48 (1.06-2.06)	2.16 (1.22-3.84)	1.64 (1.02-2.63)	0.82 (0.54-1.25)	0.91 (0.53-1.54)	1.11 (0.93-1.33)	0.65 (0.47-0.88)	1.80 (1.13-2.85)	1.68 (0.86-3.29)	1.60 (0.68-3.78)
Decreased engagement	1.99 (1.57-2.52)	1.74 (1.33-2.27)	2.06 (1.47-2.89)	0.60 (0.43-0.83)	0.59 (0.41-0.85)	1.13 (0.94-1.36)	0.44 (0.35-0.55)	2.33 (1.72-3.15)	3.53 (2.46-5.06)	8.62 (4.41-16.85)
Consistent nonparticipation	1.50 (1.05-2.15)	2.30 (1.56-3.39)	2.53 (1.63-3.94)	0.89 (0.56-1.42)	0.39 (0.24-0.64)	1.59 (0.88-2.84)	0.42 (0.30-0.59)	2.81 (1.81-4.37)	3.42 (1.93-6.09)	8.49 (3.59-20.11)
Model 1^c^										
Increased engagement	1.21 (0.87-1.68)	1.18 (0.70-1.99)	1.27 (0.78-2.05)	0.86 (0.56-1.32)	1.11 (0.65-1.90)	1.08 (0.91-1.27)	1.10 (0.79-1.53)	1.28 (0.81-2.01)	1.20 (0.56-2.58)	4.02 (0.75-21.44)
Decreased engagement	1.56 (1.21-2.00)	1.30 (0.96-1.75)	1.51 (1.08-2.12)	0.63 (0.46-0.88)	0.69 (0.46-1.05)	1.16 (0.96-1.40)	0.61 (0.47-0.80)	1.49 (1.10-2.02)	2.00 (1.39-2.88)	1.41 (0.72-2.78)
Consistent nonparticipation	1.04 (0.72-1.50)	1.33 (0.90-1.97)	1.60 (1.00-2.56)	0.98 (0.61-1.58)	0.56 (0.33-0.94)	1.68 (0.91-3.09)	0.88 (0.61-1.28)	1.62 (0.99-2.65)	1.79 (0.94-3.39)	3.17 (0.87-11.52)
Model 2^d^										
Increased engagement	1.02 (0.73-1.42)	0.93 (0.56-1.53)	0.98 (0.60-1.61)	0.78 (0.52-1.19)	0.91 (0.52-1.57)	0.92 (0.79-1.07)	1.20 (0.86-1.68)	0.98 (0.61-1.56)	0.95 (0.43-2.12)	2.82 (0.67-11.83)
Decreased engagement	1.44 (1.11-1.86)	1.23 (0.90-1.67)	1.34 (0.96-1.89)	0.60 (0.43-0.83)	0.60 (0.40-0.89)	1.02 (0.85-1.22)	0.64 (0.49-0.84)	1.29 (0.95-1.77)	1.78 (1.23-2.57)	1.61 (0.71-3.64)
Consistent nonparticipation	0.90 (0.61-1.31)	1.10 (0.71-1.71)	1.31 (0.80-2.13)	0.90 (0.56-1.45)	0.49 (0.29-0.83)	1.22 (0.83-1.79)	0.96 (0.65-1.41)	1.29 (0.78-2.12)	1.52 (0.78-2.98)	5.11 (1.07-24.37)
**Physical activities** ^g^										
Crude model										
Increased engagement	1.41 (1.13-1.74)	2.11 (1.48-3.01)	1.83 (1.36-2.46)	1.01 (0.79-1.28)	0.92 (0.62-1.39)	1.14 (0.98-1.32)	0.47 (0.39-0.58)	1.70 (1.24-2.32)	2.07 (1.38-3.10)	3.35 (1.73-6.49)
Decreased engagement	1.82 (1.53-2.17)	2.25 (1.72-2.93)	1.99 (1.54-2.56)	1.06 (0.87-1.29)	0.66 (0.50-0.88)	1.28 (1.07-1.53)	0.44 (0.37-0.52)	2.17 (1.69-2.79)	2.40 (1.74-3.32)	6.49 (3.55-11.85)
Consistent nonparticipation	1.77 (1.46-2.14)	2.29 (1.77-2.97)	2.46 (1.89-3.18)	0.93 (0.74-1.17)	0.93 (0.66-1.31)	1.25 (1.11-1.40)	0.34 (0.28-0.41)	2.43 (1.88-3.14)	3.02 (2.18-4.19)	4.56 (2.39-8.71)
Model 1^c^										
Increased engagement	1.25 (1.01-1.56)	1.48 (1.10-2.00)	1.56 (1.16-2.10)	1.08 (0.84-1.38)	1.13 (0.74-1.73)	1.13 (0.99-1.30)	0.61 (0.49-0.76)	1.44 (1.06-1.97)	1.71 (1.08-2.71)	8.26 (3.36-20.27)
Decreased engagement	1.58 (1.32-1.89)	1.99 (1.55-2.57)	1.63 (1.25-2.12)	1.14 (0.93-1.39)	0.80 (0.59-1.09)	1.30 (1.10-1.54)	0.58 (0.48-0.69)	1.75 (1.35-2.27)	1.74 (1.24-2.44)	2.79 (1.60-4.87)
Consistent nonparticipation	1.42 (1.16-1.73)	1.69 (1.29-2.20)	1.84 (1.41-2.39)	0.99 (0.79-1.25)	1.13 (0.79-1.62)	1.23 (1.10-1.39)	0.50 (0.41-0.60)	1.82 (1.38-2.39)	2.09 (1.48-2.95)	2.59 (1.22-5.50)
Model 2^d^										
Increased engagement	1.09 (0.87-1.35)	1.33 (0.96-1.84)	1.27 (0.95-1.72)	1.01 (0.78-1.30)	0.99 (0.64-1.53)	1.03 (0.90-1.18)	0.63 (0.51-0.79)	1.19 (0.88-1.62)	1.48 (0.93-2.37)	5.58 (2.21-14.09)
Decreased engagement	1.40 (1.16-1.68)	1.82 (1.40-2.36)	1.40 (1.07-1.82)	1.08 (0.88-1.32)	0.69 (0.51-0.95)	1.14 (1.01-1.29)	0.60 (0.50-0.72)	1.52 (1.16-1.99)	1.56 (1.11-2.20)	3.02 (1.66-5.49)
Consistent nonparticipation	1.14 (0.93-1.40)	1.34 (1.04-1.74)	1.41 (1.07-1.86)	0.90 (0.71-1.14)	0.90 (0.63-1.29)	1.04 (0.93-1.18)	0.54 (0.44-0.66)	1.42 (1.06-1.90)	1.70 (1.18-2.45)	2.34 (1.00-5.47)

Abbreviations: IRR, incidence rate; OR, odds ratio.

^a^ All analyses were based on weighted and imputed data sets. The consistent engagement group was the referenced group.

^b^ Community activities included volunteering, charity work, educational courses, sports or social clubs, and meetings of nonreligious organizations.

^c^ Model 1 adjusted for predisposing and enabling factors (ie, age, gender, educational level, race and ethnicity, marital status, employment or occupation, household wealth, and life satisfaction).

^d^ Model 2 adjusted for confounders in model 1 and health-related covariates (ie, depression, multimorbidity, activities of daily living, instrumental activities of daily living, and self-rated health status).

^e^ Cognitive activities included reading, word games, cards, and writing.

^f^ Home-based creative activities included gardening, baking, cooking, crafts, and hobbies.

^g^ Physical activities included exercise and walking.

#### Outpatient Care

Decreased engagement in overall SCCE was associated with lower odds of physician visits (OR, 0.68; 95% CI, 0.50-0.93). Similarly, consistent nonparticipation was associated with lower odds of outpatient surgery (OR, 0.78; 95% CI, 0.66-0.93), physician visits (OR, 0.62; 95% CI, 0.46-0.82), and a decreased relative number of physician visits (IRR, 0.91; 95% CI, 0.83-0.99) (Table 4). These associations were driven by decreased and nonparticipation in community, creative, and physical activities (Table 5).

#### Dental Care

Decreased and nonparticipation in SCCE were associated with lower odds of dental care utilization (decreased engagement: OR, 0.68; 95% CI, 0.57-0.81; nonparticipation: OR, 0.51; 95% CI, 0.44-0.60) (Table 4). These associations were driven by decreased and nonparticipation in community, creative, and physical activities. However, increased engagement in community or physical activities was also associated with lower odds of dental care utilization (Table 5).

#### Community Health Care

In the fully adjusted models, there was no evidence for associations between changes in overall SCCE and community health care utilization (Table 4). However, when looking at specific types of activities, increased community engagement was associated with lower odds of using community health care, while decreased or nonparticipation in creative and physical activities were associated with increased usage (Table 5).

## Discussion

In this cohort study of a nationally representative sample of older adults in the US, those with more frequent SCCE used less inpatient care and community health care but made greater use of outpatient and dental care, independent of sociodemographic and health-related confounders. Longitudinal analyses broadly confirm these findings, showing that consistent nonparticipation or reduced engagement over time was associated with higher subsequent utilization of inpatient hospital care and community health care as well as lower utilization of outpatient and dental care. However, there was some variation in results by different types of activities, which could be due to different mechanisms underpinning associations.

### Inpatient and Community Health Care

SCCE was associated with reduced length of hospital stays and reduced odds of nursing home stays and home health care utilization. These types of health care depend on physical function and capacity for individuals to be discharged home, suggesting that SCCE may support the maintenance of such function. Indeed, the associations were most consistent for engagement in physical and creative activities, which include activities most intrinsic to independent living (eg, walking, cooking, gardening, and hobbies, which can include do-it-yourself–related or textile-based projects). This finding echoes research showing protective associations of physical and creative activities with age-related decline, including the onset and progression of frailty, chronic pain, and age-related disability.^43,44^ The short-term findings were supported by our longitudinal analyses, as decreased engagement and consistent nonparticipation in creative and physical activities were associated with increased length of hospital stay and (for physical activities) use of community health care. This is also in line with previous evidence on the associations of physical activity with lower levels of health care utilization in later life, which could be explained through the health benefits of physical activity, including improved physical functional abilities, facilitated disease recovery, and phycological benefits.^45,46,47^

Our results are consistent with previous theories that SCCE could reduce demand for secondary care services,^24^ although they show nuance in 2 ways. First, it was length of hospital stay rather than admissions or readmissions that were associated with SCCE. This supports previous evidence that hobbies and volunteering were associated with reduced home health care utilization but not hospital admissions.^31^ It also supports work on broader social connections, which has similarly found inconsistent or null findings for hospital admissions but significant associations of smaller social network size and lower perceived support with increased length of time spent in hospital.^25^ Thus, SCCE may be mechanistically associated with length of hospital stay via enhancing social networks and support.^48^ Our findings are different from those reported in an observational study by Bu et al^49^ that found that low SCCE was associated with increased risk of hospitalization for respiratory events, but the specificity of the outcome measure may have influenced the findings. Future research should focus on subtypes of hospital admissions and different at-risk groups. Second, we did not see associations for increased SCCE, just for reduced engagement or nonparticipation. This suggests that the associations of such engagement with changes in health care utilization may mainly derive from regular SCCE in the long term rather than short-term behavior change. The potential outcomes associated with such SCCE are about maintaining existing function rather than increasing that function, akin to a “use or lose it” mechanism. This echoes previous evidence on the dose-response association between SCCE (eg, physical activity) and health care utilization.^50^ Although our findings support evidence on inpatient health care utilization from previous evaluations of social prescribing initiatives that refer people to SCCE, it may be that these activities were enabling people to maintain existing SCCE rather than introducing entirely new activities into people’s lives.^28,29,30^ This remains to be explored further in future intervention studies.

### Outpatient and Dental Care

SCCE was associated during the 2-year follow-up period with higher outpatient surgery and dental care utilization, while longitudinally, decreased and nonparticipation in SCCE were associated with decreased utilization of both. A delay in health care seeking is a key driver of accumulated unmet health needs, which could hinder early detection and preventive care for disease progression and increase the risk of high health care expenditure, especially in aging populations.^51,52^ Therefore, this finding is of particular interest. Notably, the association was found across multiple different types of SCCE, and the findings for dental care echo a cross-sectional analysis by Burr and Lee^32^ of the same data set focusing specifically on religious attendance, which could be considered another broader form of SCCE. One key mechanistic candidate that could explain the association across all types of SCCE is patient activation, in which individuals who show greater SCCE, indicating greater motivation, proactivity, and self-efficacy, may have more knowledge, skills, and confidence for managing their health.^53^ Previous evidence has shown that positive patient activation is associated with a range of healthy self-management behaviors,^54^ as well as specific patterns of health care utilization that support the findings of this study, including increased elective rather than emergency health care engagement.^55^

We also found increased physician visits among people with greater cognitive and community activities, with the pattern replicated longitudinally, as participants with decreased engagement and nonparticipation in SCCE over time had decreased physician visits. This echoes findings from a study on hobbies and volunteering by Brasher and Leedahl,^31^ which found a short-term association of SCCE with increased physician visits. These findings could reflect a social capital effect, in that people who engage with others in the community may have greater awareness of when and how to access health care.^56^ However, it is unlikely to be purely driven by this mechanism, as a systematic review of social relationships and health care utilization by Valtorta et al^25^ found inconsistent results regarding the enablement or prevention of physician contact. Other mechanisms, such as increased health literacy providing enhanced understanding and capacity to manage primary care engagement, may also be key.^18^ However, there was evidence in our study of a possible inverted U shape in this association, with increases in community and cognitive engagement over time also associated with fewer physician visits. This is not surprising, given that there is a similar inverted *U* pattern in primary care contacts seen by patient activation levels.^55^ But it may also reflect that individuals who took up more activities experienced better health and reduced need to access health care, potentially in a bidirectional loop.

### Limitations

This study has several limitations. Our findings may be biased by the measures used in HRS. The outcomes and exposures were collected through participants’ recall at baseline and follow-up, which may be subject to recall bias. We used data-driven categories of SCCE, but there is considerable overlap and shared ingredients among such activities that need to be acknowledged; artificial compartmentalization of activities may mask overlapping behavioral patterns across different activities in individuals. We measured changes in the frequency of SCCE instead of calculating change in SCCE scores due to statistical issues with change scores,^57^ but more work is needed to explore changes in engagement over time for more precise estimates. A large number of comparisons of the associations between SCCE and health care utilization is another potential limitation. Although confounders were selected based on previous theoretical models and literature, we cannot rule out the possibility of residual confounding. For example, the absence of cognitive function measures may bias estimates. Meanwhile, some of the included confounders may mediate the associations, leading to overadjustment bias. In the longitudinal analyses, relatively healthier participants might be more likely to be involved in SCCE activities and be successfully followed up, which may lead to selection bias and the possibility of reverse causality in our estimates. Additionally, we accounted for the covariates at baseline, which might result in an overestimation of associations. Therefore, this observational analysis is not able to identify a causal relationship between SCCE and health care utilization. Further experimental studies focusing on repeated measures of SCCE and clinically validated health-seeking behavior should explore the interplay between these behaviors in more detail and the potential mechanisms linking them. Beyond that, the role of SCCE in different types of disease management and treatment requires further investigation to best meet the complex health needs of an aging society.

## Conclusions

This cohort study found an association between SCCE and health care utilization, as SCCE had protective associations with changing patterns of health care utilization, including lower inpatient and community health care utilization and greater outpatient and dental care interactions. These associations were mainly independent of demographic, socioeconomic, and health-related confounders. Although lower levels of SCCE and health care utilization both tend to be more concentrated in individuals from lower socioeconomic backgrounds,^23,58^ SCCE may be associated with health care utilization independent of socioeconomic position. Our results, in particular our comparisons of different types of SCCE, shed light on potential mechanisms underpinning the association. Our longitudinal findings also indicate the importance of maintaining SCCE in older age, as decreased engagement and nonparticipation in SCCE had adverse associations with health care utilization. Our findings support further experimental research on the mechanisms underlying greater participation in SCCE and health care utilization, which could include supporting health outcomes and facilitating preventive health care–seeking behaviors. SCCE has the potential to act as a pragmatic health promotion approach to shape beneficial health-seeking behaviors, which could lead to potential health benefits, such as early detection and preventive care. Promoting SCCE could support the decentralization of health care systems through addressing more health needs in the early stage of diseases, supporting primary care delivery, and reducing inpatient and community health care utilization, resulting in alleviated health care costs both at the individual level and in long-term health and social care. The reality of this potential remains to be explored further through in-depth evaluations of social prescribing within health and social care systems using a systems approach.

## Notes

Supplement 1. **eFigure 1.** Selection of the Study Sample **eFigure 2.** Spaghetti Plots for the Mean Level of Overall SCCE Among Longitudinal Study Samples in Different Starting Points **eFigure 3.** Spaghetti Plots for the Mean Level of Specific Subtypes of SCCE Among Longitudinal Study Samples in Different Starting Points **eTable 1.** Exploratory Factor Analysis of the Items of Social, Cultural, and Community Engagement **eTable 2.** Percentage of Missingness for Variables Among Study Participants **eTable 3.** Associations Between Overall SCCE and Subsequent Health Care Utilization at Baseline in the Short-term Analysis **eTable 4.** Associations Between Specific Subtypes of SCCE and Subsequent Health Care Utilization at Baseline in the Short-term Analysis **eTable 5.** Associations Between Changes in Overall SCCE and Subsequent Health Care Utilization in the Longitudinal Analysis **eTable 6.** Associations Between Specific Subtypes of SCCE and Subsequent Health Care Utilization in the Longitudinal Analysis

Supplement 2.
Data Sharing Statement
