# Supplementary material for: Associations of Social, Cultural, and Community Engagement With Health Care Utilization in the US Health and Retirement Study
Source: JAMA Netw Open. 2023 Apr 4;6(4):e236636. doi: 10.1001/jamanetworkopen.2023.6636 (PMC10074222; doi:10.1001/jamanetworkopen.2023.6636)
Supplement: Supplement 1. — eFigure 1. Selection of the Study Sample eFigure 2. Spaghetti Plots for the Mean Level of Overall SCCE Among Longitudinal Study Samples in Different Starting Points eFigure 3. Spaghetti Plots for the Mean Level of Specific Subtypes of SCCE Among Longitudinal Study Samples in Different Starting Points eTable 1. Exploratory Factor Analysis of the Items of Social, Cultural, and Community Engagement eTable 2. Percentage of Missingness for Variables Among Study Participants eTable 3. Associations Between Overall SCCE and Subsequent Health Care Utilization at Baseline in the Short-term Analysis eTable 4. Associations Between Specific Subtypes of SCCE and Subsequent Health Care Utilization at Baseline in the Short-term Analysis eTable 5. Associations Between Changes in Overall SCCE and Subsequent Health Care Utilization in the Longitudinal Analysis eTable 6. Associations Between Specific Subtypes of SCCE and Subsequent Health Care Utilization in the Longitudinal Analysis [file jamanetwopen-e236636-s001.pdf]

Supplemental Online Content

Gao Q, Bone JK, Bu F, Paul E, Sonke JK, Fancourt D. Associations of social, cultural, and community engagement with health care utilization in the US Health and Retirement Study. *JAMA Netw Open*. 2023;6(4):e236636. doi:10.1001/jamanetworkopen.2023.6636

eFigure 1. Selection of the Study Sample

eFigure 2. Spaghetti Plots for the Mean Level of Overall SCCE Among Longitudinal Study Samples in Different Starting Points

eFigure 3. Spaghetti Plots for the Mean Level of Specific Subtypes of SCCE Among Longitudinal Study Samples in Different Starting Points

eTable 1. Exploratory Factor Analysis of the Items of Social, Cultural, and Community Engagement

eTable 2. Percentage of Missingness for Variables Among Study Participants

eTable 3. Associations Between Overall SCCE and Subsequent Health Care Utilization at Baseline in the Short-term Analysis

eTable 4. Associations Between Specific Subtypes of SCCE and Subsequent Health Care Utilization at Baseline in the Short-term Analysis

eTable 5. Associations Between Changes in Overall SCCE and Subsequent Health Care Utilization in the Longitudinal Analysis

eTable 6. Associations Between Specific Subtypes of SCCE and Subsequent Health Care Utilization in the Longitudinal Analysis

This supplemental material has been provided by the authors to give readers additional information about their work.

© 2023 Gao Q et al. *JAMA Network Open.*

eFigure 1. Selection of the Study Sample

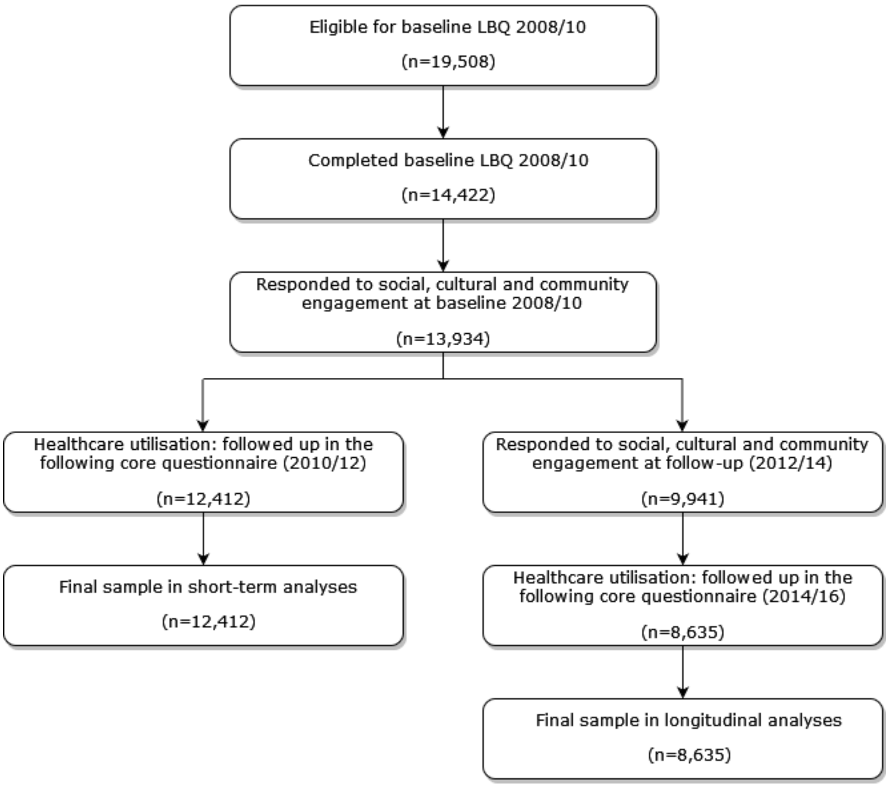

© 2023 Gao Q et al. *JAMA Network Open.*

eFigure 2. Spaghetti Plots for the Mean Level of Overall SCCE Among Longitudinal Study Samples in Different Starting Points

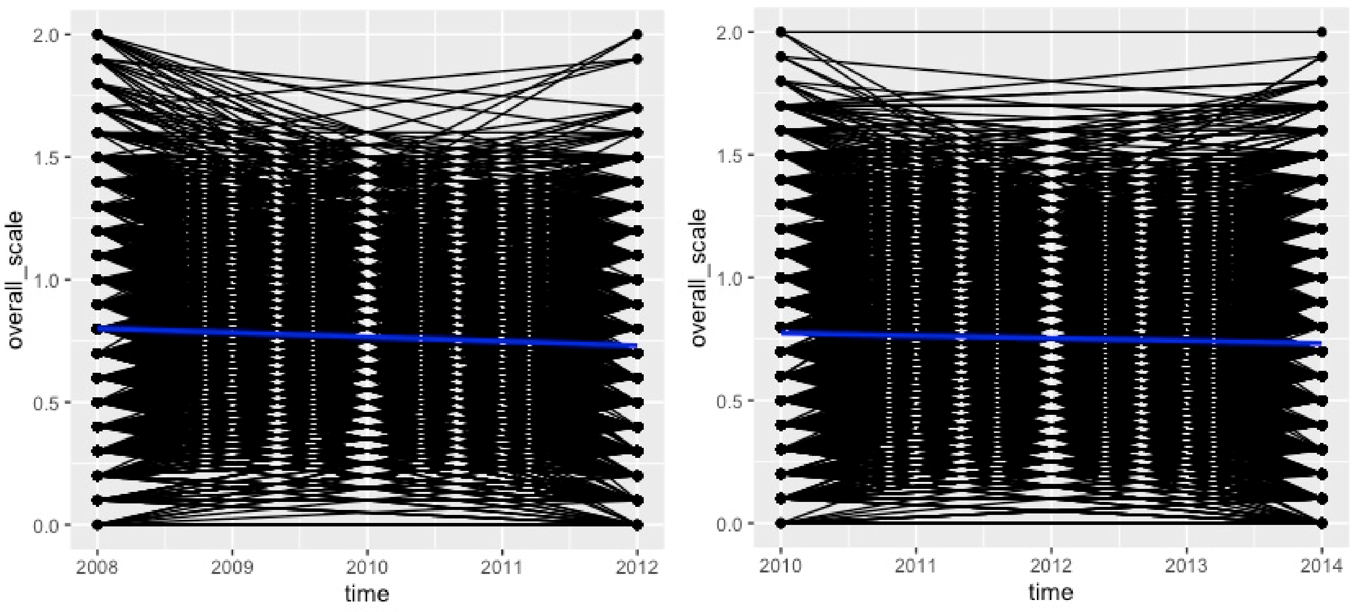

(a) overall scale (2008/12)

(b) overall scale (2010/14)

© 2023 Gao Q et al. *JAMA Network Open.*

eFigure 3. Spaghetti Plots for the Mean Level of Specific Subtypes of SCCE Among Longitudinal Study Samples in Different Starting Points

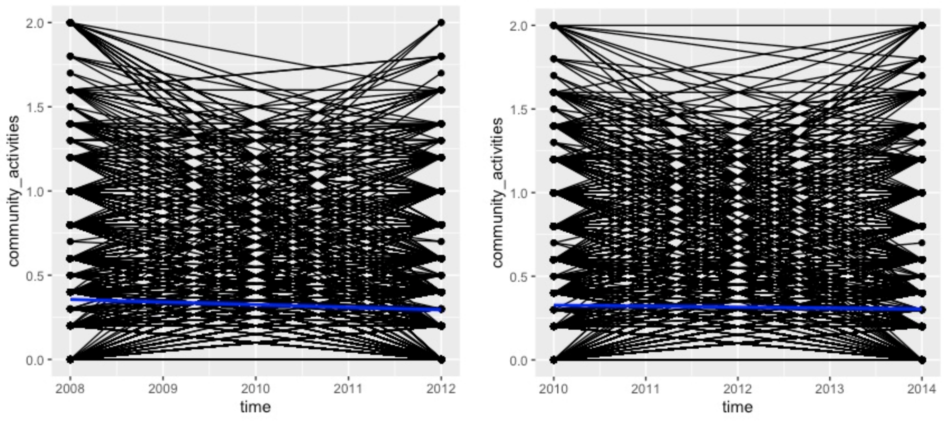

(a) community activities (2008/12 and 2010/14)

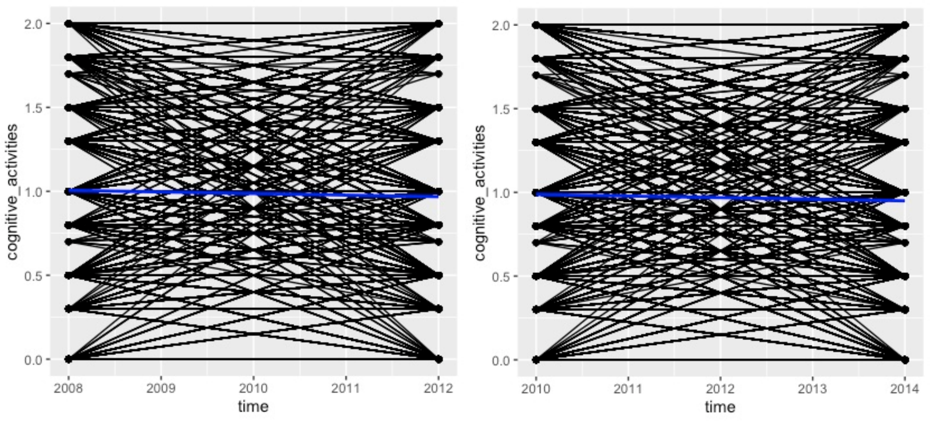

(b) cognitive activities (2008/12 and 2010/14)

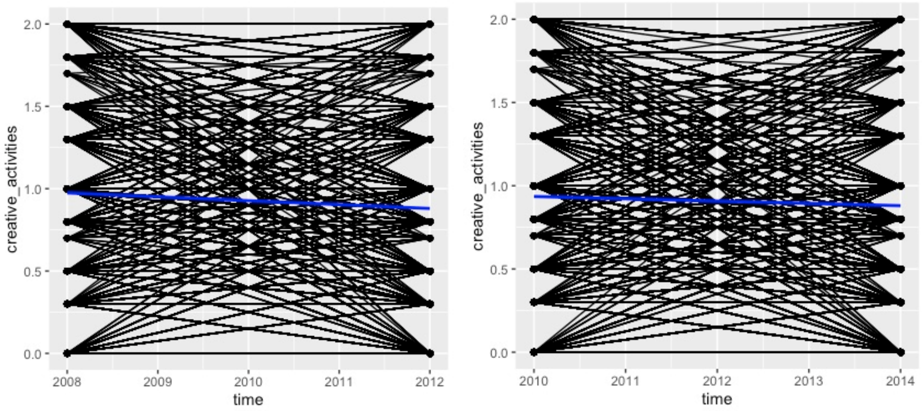

(c) creative activities (2008/12 and 2010/14)

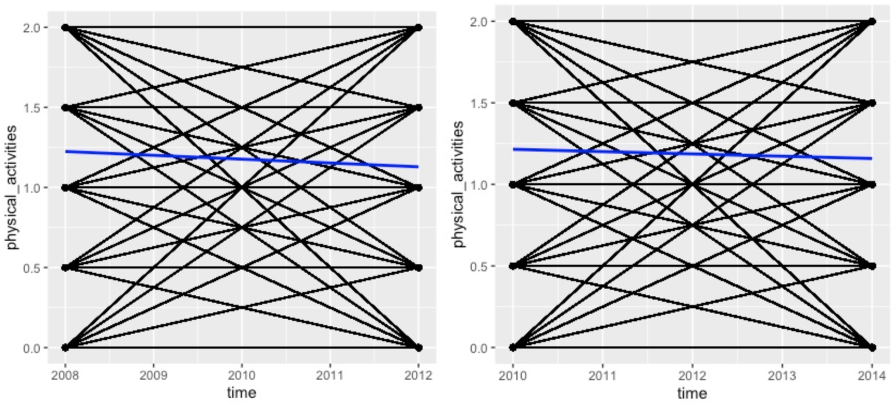

(d) physical activities (2008/12 and 2010/14)

© 2023 Gao Q et al. *JAMA Network Open.*

eTable 1. Exploratory Factor Analysis of the Items of Social, Cultural, and Community Engagement

Items	Factor				Dimension
	1	2	3	4	
1. Do volunteer work with children or young people?	**0.49**	0.07	0.15	-0.01	**Community activities**
2. Do any other volunteer or charity work?	**0.57**	0.13	0.11	0.05	
3. Attend an educational or training course?	**0.52**	0.14	0.11	0.03	
4. Go to a sport, social, or other club?	**0.37**	0.31	-0.01	0.21	
5. Attend meetings of non-religious organisations, such as political, community, or other interest groups?	**0.55**	0.11	0.07	0.13	
6. Read books, magazines, or newspapers?	0.10	0.23	0.16	**0.31**	**Cognitive activities**
7. Do word games such as crossword puzzles or Scrabble?	0.06	0.04	0.16	**0.49**	
8. Play cards or games such as chess?	0.14	0.06	0.10	**0.42**	
9. Do writing (such as letters, stories, or journal entries)?	0.32	0.16	0.25	**0.23**	
10. Do home or car maintenance or gardening?	0.06	0.37	**0.35**	-0.02	**Home-based creative activities**
11. Bake or cook something special?	0.05	0.16	**0.42**	0.13	
12. Make clothes, knit, embroider, etc.?	0.20	-0.01	**0.45**	0.18	
13. Work on a hobby or project?	0.25	0.25	**0.46**	0.13	
14. Play sports or exercise?	0.17	**0.63**	0.06	0.08	**Physical activities**
15. Walk for 20 minutes or more?	0.08	**0.58**	0.11	-0.00	

Note. To explore the factorial structure of 15-items of social, cultural and community engagement based on based on 2008/10 waves (n=12,625). Maximum likelihood (ML) extraction was used to estimate the model. Model fit: Likelihood ratio x^2 =4739.402 (p<0.001), Root mean squared error of approximation (RMSEA)= 0.066, Standardized root mean squared residual (SRMR)= 0.048, Akaike's information criterion (AIC)= 691977.545, Bayesian information criterion (BIC) = 692357.160, Comparative fit index (CFI) =0.843, Tucker-Lewis index (TLI)= 0.804

© 2023 Gao Q et al. *JAMA Network Open.*

eTable 2. Percentage of Missingness for Variables Among Study Participants

**Characteristics**	**% (Short-term sample, n=12,412)**	**% (Longitudinal sample, n=8,635)**
Age (in years)	0%	0%
Gender	0%	0%
Race/Ethnicity	<0.1%	<0.1%
Marital status	<0.1%	<0.1%
Education	0.3%	0.3%
Employment/occupation	0.4%	0.5%
Household wealth [quintiles]	0%	0%
Life satisfaction	1.9%	1.0%
Self-rated health status	<0.1%	<0.1%
ADLs	0%	0%
IADLs	0%	0%
Multimorbidity	<0.1%	<0.1%
Depression	1.7%	0.9%
Overall	2.6%	1.8%

© 2023 Gao Q et al. *JAMA Network Open.*

Complete case analyses

eTable 3. Associations Between Overall SCCE and Subsequent Health Care Utilization at Baseline in the Short-term Analysis

**Outcomes**	**Inpatient care**			**Outpatient care**			**Dental care**	**Community healthcare**		
	Hospital stays (OR)	Length of hospital stay (IRR)	Readmission to hospital (2-year) (OR)	Outpatient surgery (OR)	Physician visits (OR)	Number of physician visits (IRR)	Dental care and dentures (OR)	Home health care (OR)	Nursing home stays (OR)	Nights in nursing home (IRR)
Crude model	0.48 (0.41-0.56)	0.40 (0.31-0.50)	0.31 (0.24-0.39)	1.28 (1.09-1.50)	2.00 (1.53-2.61)	0.94 (0.83-1.06)	4.50 (3.83-5.27)	0.32 (0.25-0.43)	0.19 (0.12-0.29)	0.22 (0.10-0.49)
Model 1	0.65 (0.55-0.76)	0.55 (0.42-0.70)	0.49 (0.39-0.63)	1.18 (0.99-1.41)	1.00 (0.77-1.32)	0.94 (0.84-1.06)	1.79 (1.51-2.11)	0.48 (0.36-0.63)	0.30 (0.19-0.47)	0.44 (0.21-0.93)
Model 2	0.87 (0.73-1.03)	0.68 (0.53-0.88)	0.77 (0.60-0.99)	1.32 (1.10-1.58)	1.28 (0.96-1.70)	1.09 (0.96-1.23)	1.71 (1.44-2.03)	0.73 (0.55-0.97)	0.49 (0.31-0.77)	0.53 (0.24-1.15)

Note. All analyses were based on weighted datasets. Odds ratios (ORs), Incidence rate ratios (IRRs) and 95% confidence intervals (CI) are presented in the table. Model 1 adjusted for predisposing and enabling factors (age, gender, educational level, race/ethnicity, marital status, employment/occupation, household wealth and life satisfaction). Model 2 adjusted for confounders in Model 1 and health-related covariates (depression, multimorbidity, ADLs, IADLs, and self-rated health status). SCCE measure: mean scores of social, cultural and community engagement within each sub-scale. Community activities (volunteering, charity work, educational courses, sports or social clubs, meetings of non-religious organisations), cognitive activities (reading, word games, cards, writing), home-based creative activities (gardening, baking, cooking, crafts, hobbies), and physical activities (exercise, walking).

© 2023 Gao Q et al. *JAMA Network Open.*

eTable 4. Associations Between Specific Subtypes of SCCE and Subsequent Health Care Utilization at Baseline in the Short-term Analysis

Outcomes	Inpatient care			Outpatient care			Dental care	Community healthcare		
	Hospital stays (OR)	Length of hospital stay (IRR)	Readmission to hospital (2-year) (OR)	Outpatient surgery (OR)	Physician visits (OR)	Number of physician visits (IRR)	Dental care and dentures (OR)	Home health care (OR)	Nursing home stays (OR)	Nights in nursing home (IRR)
**Community activities**										
Crude model	0.70 (0.61-0.81)	0.75 (0.61-0.92)	0.64 (0.51-0.80)	1.10 (0.96-1.27)	1.60 (1.24-2.07)	1.08 (0.98-1.20)	2.68 (2.30-3.12)	0.69 (0.54-0.89)	0.43 (0.29-0.64)	0.48 (0.24-0.98)
Model 1	0.87 (0.76-1.01)	0.96 (0.77-1.20)	0.88 (0.71-1.09)	1.04 (0.90-1.21)	1.07 (0.84-1.37)	1.13 (1.02-1.25)	1.48 (1.28-1.71)	0.90 (0.71-1.14)	0.61 (0.42-0.89)	0.79 (0.39-1.63)
Model 2	0.98 (0.85-1.13)	1.04 (0.83-1.29)	1.05 (0.85-1.30)	1.08 (0.92-1.25)	1.18 (0.92-1.50)	1.18 (1.07-1.29)	1.44 (1.25-1.67)	1.07 (0.84-1.35)	0.75 (0.51-1.09)	0.79 (0.38-1.65)
**Cognitive activities**										
Crude model	0.94 (0.85-1.04)	0.88 (0.73-1.06)	0.85 (0.73-0.99)	1.21 (1.08-1.35)	1.69 (1.39-2.07)	1.14 (1.04-1.25)	1.65 (1.49-1.82)	0.83 (0.70-0.98)	0.97 (0.74-1.29)	0.86 (0.57-1.31)
Model 1	0.98 (0.88-1.09)	0.91 (0.77-1.08)	0.94 (0.80-1.10)	1.14 (1.01-1.28)	1.11 (0.91-1.35)	1.08 (1.00-1.18)	1.11 (1.00-1.24)	0.80 (0.68-0.96)	0.93 (0.71-1.23)	1.52 (0.89-2.60)
Model 2	1.07 (0.96-1.20)	0.93 (0.79-1.10)	1.09 (0.92-1.28)	1.17 (1.03-1.32)	1.17 (0.96-1.42)	1.10 (1.02-1.19)	1.09 (0.97-1.22)	0.93 (0.78-1.11)	1.13 (0.86-1.50)	2.02 (1.23-3.31)
**Creative activities**										
Crude model	0.65 (0.58-0.71)	0.56 (0.48-0.65)	0.50 (0.43-0.58)	1.15 (1.04-1.28)	1.19 (1.02-1.38)	0.91 (0.84-0.98)	1.77 (1.61-1.94)	0.48 (0.40-0.57)	0.27 (0.20-0.36)	0.31 (0.19-0.50)
Model 1	0.78 (0.70-0.86)	0.73 (0.63-0.84)	0.67 (0.58-0.77)	1.12 (1.01-1.26)	0.89 (0.75-1.05)	0.91 (0.84-0.98)	1.16 (1.04-1.28)	0.66 (0.56-0.77)	0.41 (0.32-0.54)	0.28 (0.18-0.43)
Model 2	0.89 (0.80-0.99)	0.82 (0.70-0.96)	0.82 (0.71-0.95)	1.18 (1.05-1.32)	1.00 (0.83-1.17)	0.97 (0.90-1.04)	1.12 (1.01-1.25)	0.81 (0.68-0.95)	0.51 (0.39-0.68)	0.37 (0.24-0.58)
**Physical activities**										

© 2023 Gao Q et al. *JAMA Network Open.*

Crude model	0.69 (0.65-0.73)	0.60 (0.54-0.67)	0.56 (0.52-0.62)	1.01 (0.94-1.08)	1.12 (1.01-1.24)	0.85 (0.81-0.89)	1.75 (1.64-1.85)	0.61 (0.55-0.67)	0.54 (0.46-0.63)	0.54 (0.39-0.74)
Model 1	0.78 (0.73-0.84)	0.68 (0.61-0.75)	0.66 (0.60-0.72)	1.00 (0.92-1.06)	0.98 (0.87-1.09)	0.88 (0.84-0.92)	1.38 (1.29-1.48)	0.71 (0.64-0.79)	0.66 (0.56-0.78)	0.72 (0.53-0.99)
Model 2	0.89 (0.83-0.96)	0.76 (0.69-0.85)	0.78 (0.71-0.87)	1.04 (0.97-1.12)	1.10 (0.98-1.23)	0.95 (0.91-1.00)	1.35 (1.26-1.45)	0.83 (0.75-0.93)	0.77 (0.64-0.93)	0.66 (0.47-0.93)

Note. All analyses were based on weighted datasets. Odds ratios (ORs), Incidence rate ratios (IRRs) and 95% confidence intervals (CI) are presented in the table. Model 1 adjusted for predisposing and enabling factors (age, gender, educational level, race/ethnicity, marital status, employment/occupation, household wealth and life satisfaction). Model 2 adjusted for confounders in Model 1 and health-related covariates (depression, multimorbidity, ADLs, IADLs, and self-rated health status). SCCE measure: mean scores of social, cultural and community engagement within each sub-scale. Community activities (volunteering, charity work, educational courses, sports or social clubs, meetings of non-religious organisations), cognitive activities (reading, word games, cards, writing), home-based creative activities (gardening, baking, cooking, crafts, hobbies), and physical activities (exercise, walking).

© 2023 Gao Q et al. *JAMA Network Open.*

eTable 5. Associations Between Changes in Overall SCCE and Subsequent Health Care Utilization in the Longitudinal Analysis

**Outcomes**	**Inpatient care**			**Outpatient care**			**Dental care**	**Community healthcare**		
	Hospital stays (OR)	Length of hospital stay (IRR)	Readmission to hospital (2-year) (OR)	Outpatient surgery (OR)	Physician visits (OR)	Number of physician visits (IRR)	Dental care and dentures (OR)	Home health care (OR)	Nursing home stays (OR)	Nights in nursing home (IRR)
**Crude OR**										
Increased engagement	1.25 (1.02-1.54)	1.53 (1.10-2.12)	1.26 (0.91-1.75)	0.91 (0.74-1.13)	0.62 (0.43-0.90)	0.93 (0.82-1.06)	0.49 (0.40-0.60)	1.15 (0.83-1.58)	0.95 (0.59-1.53)	1.28 (0.56-2.95)
Decreased engagement	1.34 (1.14-1.58)	1.99 (1.51-2.62)	1.69 (1.31-2.17)	0.96 (0.81-1.14)	0.59 (0.44-0.79)	1.06 (0.95-1.18)	0.47 (0.40-0.55)	1.56 (1.21-2.02)	1.77 (1.27-2.47)	3.37 (1.82-6.25)
Consistent non-participation	1.49 (1.29-1.72)	2.12 (1.73-2.60)	2.07 (1.67-2.58)	0.76 (0.65-0.89)	0.46 (0.36-0.59)	1.01 (0.91-1.12)	0.27 (0.23-0.31)	1.93 (1.55-2.40)	1.73 (1.28-2.33)	2.92 (1.62-5.25)
**Model 1**										
Increased engagement	1.15 (0.93-1.42)	1.22 (0.88-1.71)	1.11 (0.80-1.53)	0.95 (0.77-1.19)	0.77 (0.53-1.11)	0.92 (0.82-1.04)	0.69 (0.56-0.85)	1.07 (0.77-1.49)	0.87 (0.52-1.45)	0.75 (0.34-1.66)
Decreased engagement	1.13 (0.96-1.34)	1.52 (1.18-1.96)	1.40 (1.08-1.81)	0.99 (0.83-1.19)	0.72 (0.53-0.98)	1.07 (0.97-1.18)	0.65 (0.55-0.77)	1.28 (0.98-1.66)	1.29 (0.92-1.81)	1.55 (0.87-2.76)
Consistent non-participation	1.15 (0.99-1.34)	1.56 (1.24-1.96)	1.50 (1.20-1.89)	0.81 (0.68-0.95)	0.69 (0.52-0.91)	1.03 (0.93-1.14)	0.49 (0.42-0.57)	1.42 (1.12-1.80)	1.13 (0.82-1.56)	1.62 (0.89-2.97)
**Model 2**										
Increased engagement	1.11 (0.90-1.37)	1.20 (0.90-1.62)	1.04 (0.75-1.44)	0.93 (0.75-1.16)	0.74 (0.51-1.07)	0.91 (0.81-1.02)	0.69 (0.56-0.85)	1.01 (0.73-1.41)	0.83 (0.49-1.40)	0.91 (0.38-2.16)
Decreased engagement	1.03 (0.87-1.23)	1.34 (1.04-1.72)	1.23 (0.95-1.59)	0.96 (0.80-1.15)	0.67 (0.49-0.92)	0.99 (0.90-1.09)	0.68 (0.57-0.80)	1.12 (0.86-1.46)	1.14 (0.81-1.61)	1.38 (0.72-2.63)
Consistent non-participation	1.01 (0.86-1.18)	1.33 (1.05-1.70)	1.26 (1.00-1.59)	0.76 (0.64-0.90)	0.60 (0.45-0.80)	0.91 (0.84-0.99)	0.52 (0.44-0.60)	1.19 (0.93-1.53)	0.97 (0.69-1.37)	1.62 (0.85-3.09)

© 2023 Gao Q et al. *JAMA Network Open.*

Note. All analyses were based on weighted datasets. Odds ratios (ORs), Incidence rate ratios (IRRs) and 95% confidence intervals (CI) are presented in the table. The consistent engagement was the referenced group. Model 1 adjusted for predisposing and enabling factors (age, gender, educational level, race/ethnicity, marital status, employment/occupation, household wealth and life satisfaction). Model 2 adjusted for confounders in Model 1 and health-related covariates (depression, multimorbidity, ADLs, IADLs, and self-rated health status).

© 2023 Gao Q et al. *JAMA Network Open.*

eTable 6. Associations Between Specific Subtypes of SCCE and Subsequent Health Care Utilization in the Longitudinal Analysis

**Outcomes**	**Inpatient care**			**Outpatient care**			**Dental care**	**Community healthcare**		
**Community activities**	Hospital stays (OR)	Length of hospital stay (IRR)	Readmission to hospital (2-year) (OR)	Outpatient surgery (OR)	Physician visits (OR)	Number of physician visits (IRR)	Dental care and dentures (OR)	Home health care (OR)	Nursing home stays (OR)	Nights in nursing home (IRR)
**Crude model**										
Increased engagement	1.24 (1.01-1.51)	1.39 (0.97-1.99)	1.06 (0.77-1.46)	0.91 (0.73-1.12)	0.77 (0.55-1.08)	0.90 (0.81-1.01)	0.53 (0.44-0.64)	1.27 (0.94-1.72)	0.67 (0.42-1.07)	0.58 (0.23-1.44)
Decreased engagement	1.25 (1.05-1.48)	1.46 (1.11-1.91)	1.47 (1.14-1.89)	0.87 (0.72-1.05)	0.76 (0.56-1.03)	1.00 (0.90-1.12)	0.51 (0.44-0.60)	1.32 (1.02-1.71)	1.55 (1.12-2.15)	2.53 (1.38-4.62)
Consistent non-participation	1.33 (1.15-1.53)	1.65 (1.34-2.03)	1.75 (1.42-2.15)	0.75 (0.64-0.88)	0.50 (0.40-0.64)	0.96 (0.86-1.08)	0.32 (0.28-0.36)	1.70 (1.38-2.10)	1.28 (0.96-1.71)	1.72 (0.93-3.18)
**Model 1**										
Increased engagement	1.14 (0.93-1.40)	1.18 (0.85-1.65)	0.93 (0.67-1.28)	0.95 (0.77-1.18)	0.97 (0.68-1.36)	0.91 (0.81-1.01)	0.74 (0.60-0.90)	1.21 (0.88-1.66)	0.60 (0.36-1.00)	0.35 (0.17-0.71)
Decreased engagement	1.07 (0.90-1.28)	1.32 (1.01-1.71)	1.24 (0.96-1.62)	0.91 (0.75-1.10)	0.99 (0.72-1.37)	1.03 (0.93-1.16)	0.73 (0.62-0.88)	1.11 (0.85-1.45)	1.18 (0.85-1.66)	1.52 (0.84-2.76)
Consistent non-participation	1.05 (0.90-1.22)	1.34 (1.07-1.68)	1.30 (1.04-1.62)	0.80 (0.68-0.94)	0.76 (0.58-0.98)	0.99 (0.88-1.11)	0.56 (0.48-0.65)	1.30 (1.03-1.64)	0.88 (0.64-1.20)	1.59 (0.87-2.91)
**Model 2**										
Increased engagement	1.08 (0.88-1.33)	1.11 (0.81-1.52)	0.86 (0.62-1.19)	0.93 (0.75-1.16)	0.92 (0.65-1.31)	0.87 (0.79-0.97)	0.76 (0.62-0.93)	1.12 (0.81-1.54)	0.55 (0.32-0.93)	0.29 (0.14-0.61)
Decreased engagement	1.00 (0.84-1.20)	1.18 (0.91-1.53)	1.13 (0.87-1.48)	0.89 (0.73-1.08)	0.95 (0.69-1.31)	0.98 (0.89-1.09)	0.76 (0.64-0.91)	1.00 (0.76-1.32)	1.06 (0.75-1.50)	1.34 (0.69-2.60)
Consistent non-participation	0.95 (0.81-1.11)	1.21 (0.95-1.54)	1.14 (0.91-1.43)	0.77 (0.65-0.91)	0.69 (0.53-0.90)	0.90 (0.82-0.98)	0.58 (0.50-0.68)	1.15 (0.90-1.46)	0.77 (0.55-1.08)	1.40 (0.73-2.68)

© 2023 Gao Q et al. *JAMA Network Open.*

Cognitive activities	Hospital stays (OR)	Length of hospital stay (IRR)	Readmission to hospital (2-year) (OR)	Outpatient surgery (OR)	Physician visits (OR)	Number of physician visits (IRR)	Dental care and dentures (OR)	Home health care (OR)	Nursing home stays (OR)	Nights in nursing home (IRR)
**Crude model**										
Increased engagement	1.39 (0.86-2.23)	1.18 (0.62-2.24)	1.62 (0.84-3.11)	0.56 (0.28-1.13)	0.29 (0.16-0.52)	0.78 (0.56-1.09)	0.30 (0.19-0.46)	1.28 (0.63-2.59)	1.06 (0.37-3.03)	0.13 (0.04-0.43)
Decreased engagement	0.92 (0.61-1.38)	1.35 (0.83-2.19)	1.58 (0.95-2.64)	1.05 (0.66-1.66)	0.49 (0.32-0.77)	1.67 (0.98-2.85)	0.37 (0.26-0.52)	1.90 (1.17-3.07)	1.54 (0.76-3.14)	2.39 (0.67-8.52)
Consistent non-participation	1.52 (0.89-2.58)	1.77 (1.05-2.98)	2.00 (1.00-4.00)	1.09 (0.59-2.02)	0.32 (0.17-0.59)	0.94 (0.62-1.41)	0.29 (0.18-0.48)	2.54 (1.31-4.90)	0.90 (0.28-2.93)	0.37 (0.09-1.59)
**Model 1**										
Increased engagement	1.19 (0.73-1.95)	0.82 (0.48-1.39)	1.27 (0.64-2.51)	0.63 (0.31-1.28)	0.50 (0.28-0.92)	0.78 (0.58-1.05)	0.59 (0.36-1.00)	1.08 (0.51-2.32)	1.06 (0.32-3.55)	0.66 (0.17-2.53)
Decreased engagement	0.76 (0.50-1.17)	1.24 (0.71-2.17)	1.23 (0.73-2.06)	1.21 (0.76-1.93)	0.86 (0.55-1.35)	1.84 (1.06-3.17)	0.78 (0.54-1.10)	1.57 (0.92-2.67)	1.29 (0.62-2.66)	0.66 (0.19-2.36)
Consistent non-participation	1.06 (0.63-1.80)	0.93 (0.51-1.70)	1.22 (0.61-2.44)	1.43 (0.75-2.73)	0.75 (0.37-1.52)	1.00 (0.67-1.50)	0.88 (0.49-1.57)	1.59 (0.81-3.12)	0.51 (0.16-1.68)	0.02 (0.004-0.06)
**Model 2**										
Increased engagement	1.12 (0.67-1.88)	0.83 (0.50-1.38)	1.13 (0.55-2.32)	0.61 (0.30-1.24)	0.49 (0.27-0.92)	0.69 (0.53-0.88)	0.61 (0.36-1.01)	0.96 (0.43-2.14)	0.95 (0.28-3.25)	1.41 (0.31-6.37)
Decreased engagement	0.63 (0.41-0.97)	1.03 (0.58-1.83)	0.96 (0.56-1.64)	1.12 (0.72-1.76)	0.75 (0.47-1.20)	1.35 (0.94-1.93)	0.84 (0.59-1.19)	1.29 (0.76-2.21)	1.11 (0.53-2.31)	0.53 (0.14-1.97)
Consistent non-participation	0.84 (0.48-1.46)	1.14 (0.56-2.33)	0.89 (0.41-1.92)	1.29 (0.66-2.51)	0.59 (0.30-1.15)	0.81 (0.57-1.16)	0.99 (0.54-1.79)	1.14 (0.56-2.33)	0.35 (0.11-1.12)	0.004 (0.001-0.02)

© 2023 Gao Q et al. *JAMA Network Open.*

**Creative activities**	Hospital stays (OR)	Length of hospital stay (IRR)	Readmission to hospital (2-year) (OR)	Outpatient surgery (OR)	Physician visits (OR)	Number of physician visits (IRR)	Dental care and dentures (OR)	Home health care (OR)	Nursing home stays (OR)	Nights in nursing home (IRR)
**Crude model**										
Increased engagement	1.44 (1.03-2.00)	2.19 (1.23-3.90)	1.67 (1.04-2.68)	0.83 (0.55-1.27)	0.89 (0.52-1.51)	1.13 (0.94-1.35)	0.64 (0.47-0.88)	1.85 (1.16-2.93)	1.71 (0.87-3.34)	1.62 (0.68-3.83)
Decreased engagement	1.99 (1.57-2.53)	1.74 (1.33-2.27)	2.12 (1.51-2.97)	0.58 (0.42-0.81)	0.57 (0.39-0.82)	1.13 (0.93-1.37)	0.44 (0.35-0.56)	2.40 (1.77-3.26)	3.64 (2.54-5.21)	8.86 (4.53-17.31)
Consistent non-participation	1.53 (1.06-2.21)	2.35 (1.58-3.50)	2.50 (1.57-3.96)	0.84 (0.52-1.36)	0.36 (0.22-0.59)	1.60 (0.86-2.96)	0.38 (0.27-0.54)	2.82 (1.79-4.46)	3.32 (1.83-6.02)	6.69 (2.65-16.92)
**Model 1**										
Increased engagement	1.18 (0.85-1.64)	1.19 (0.71-1.99)	1.29 (0.80-2.10)	0.87 (0.57-1.34)	1.07 (0.63-1.83)	1.09 (0.92-1.28)	1.10 (0.78-1.54)	1.34 (0.85-2.10)	1.25 (0.58-2.68)	3.92 (0.77-20.05)
Decreased engagement	1.57 (1.22-2.02)	1.27 (0.95-1.71)	1.57 (1.11-2.20)	0.61 (0.44-0.86)	0.67 (0.45-1.02)	1.16 (0.95-1.41)	0.62 (0.48-0.81)	1.54 (1.14-2.10)	2.09 (1.45-3.02)	1.45 (0.73-2.88)
Consistent non-participation	1.06 (0.73-1.54)	1.36 (0.91-2.02)	1.58 (0.97-2.58)	0.91 (0.56-1.49)	0.51 (0.30-0.87)	1.68 (0.88-3.20)	0.79 (0.54-1.15)	1.67 (1.00-2.76)	1.82 (0.93-3.54)	3.37 (0.90-12.57)
**Model 2**										
Increased engagement	0.99 (0.71-1.37)	0.92 (0.56-1.52)	1.00 (0.61-1.65)	0.79 (0.52-1.20)	0.87 (0.51-1.51)	0.92 (0.79-1.08)	1.19 (0.85-1.67)	1.04 (0.65-1.65)	1.01 (0.46-2.23)	2.87 (0.70-11.83)
Decreased engagement	1.44 (1.11-1.87)	1.20 (0.89-1.64)	1.39 (0.98-1.95)	0.58 (0.41-0.81)	0.57 (0.38-0.86)	1.01 (0.84-1.21)	0.65 (0.49-0.85)	1.34 (0.98-1.84)	1.86 (1.29-2.69)	1.62 (0.71-3.69)
Consistent non-participation	0.90 (0.61-1.33)	1.10 (0.71-1.71)	1.27 (0.76-2.12)	0.83 (0.51-1.36)	0.44 (0.26-0.74)	1.18 (0.78-1.79)	0.85 (0.58-1.26)	1.32 (0.79-2.21)	1.55 (0.77-3.13)	5.33 (1.10-25.80)
**Physical activities**	Hospital stays (OR)	Length of hospital stay (IRR)	Readmission to hospital (2-year) (OR)	Outpatient surgery (OR)	Physician visits (OR)	Number of physician visits (IRR)	Dental care and dentures (OR)	Home health care (OR)	Nursing home stays (OR)	Nights in nursing home (IRR)

© 2023 Gao Q et al. *JAMA Network Open.*

**Crude model**										
Increased engagement	1.39 (1.12-1.73)	2.11 (1.47-3.03)	1.88 (1.40-2.53)	0.97 (0.76-1.24)	0.98 (0.65-1.47)	1.15 (0.99-1.33)	0.48 (0.40-0.59)	1.76 (1.28-2.41)	2.10 (1.40-3.15)	3.36 (1.74-6.51)
Decreased engagement	1.82 (1.53-2.17)	2.22 (1.70-2.92)	1.99 (1.54-2.56)	1.06 (0.87-1.29)	0.66 (0.49-0.87)	1.27 (1.07-1.52)	0.45 (0.38-0.53)	2.19 (1.70-2.82)	2.37 (1.71-3.28)	6.29 (3.41-11.59)
Consistent non-participation	1.77 (1.46-2.15)	2.31 (1.77-3.01)	2.43 (1.87-3.17)	0.93 (0.74-1.17)	0.91 (0.65-1.28)	1.25 (1.12-1.41)	0.34 (0.28-0.40)	2.45 (1.89-3.18)	2.99 (2.15-4.15)	3.86 (2.02-7.36)
**Model 1**										
Increased engagement	1.24 (0.99-1.54)	1.46 (1.08-1.97)	1.60 (1.19-2.16)	1.03 (0.80-1.33)	1.20 (0.78-1.83)	1.15 (1.00-1.32)	0.63 (0.51-0.78)	1.49 (1.09-2.03)	1.74 (1.10-2.75)	8.13 (3.31-19.95)
Decreased engagement	1.58 (1.32-1.89)	2.02 (1.56-2.61)	1.64 (1.25-2.13)	1.14 (0.93-1.40)	0.80 (0.59-1.08)	1.30 (1.09-1.54)	0.59 (0.49-0.71)	1.77 (1.36-2.30)	1.72 (1.22-2.42)	2.57 (1.46-4.53)
Consistent non-participation	1.43 (1.17-1.75)	1.72 (1.32-2.25)	1.83 (1.40-2.40)	0.99 (0.78-1.25)	1.11 (0.78-1.58)	1.24 (1.10-1.40)	0.50 (0.40-0.60)	1.86 (1.41-2.45)	2.11 (1.49-3.00)	2.55 (1.20-5.41)
**Model 2**										
Increased engagement	1.08 (0.86-1.34)	1.34 (0.96-1.86)	1.31 (0.97-1.76)	0.97 (0.75-1.25)	1.04 (0.68-1.60)	1.04 (0.91-1.19)	0.65 (0.52-0.81)	1.24 (0.91-1.68)	1.52 (0.95-2.42)	5.51 (2.17-13.96)
Decreased engagement	1.40 (1.16-1.68)	1.84 (1.42-2.39)	1.40 (1.07-1.83)	1.08 (0.88-1.32)	0.69 (0.50-0.94)	1.13 (1.00-1.29)	0.61 (0.51-0.74)	1.54 (1.18-2.02)	1.56 (1.10-2.20)	2.86 (1.55-5.27)
Consistent non-participation	1.15 (0.94-1.42)	1.36 (1.05-1.76)	1.41 (1.07-1.87)	0.90 (0.71-1.14)	0.87 (0.61-1.25)	1.05 (0.93-1.19)	0.53 (0.44-0.65)	1.46 (1.09-1.96)	1.75 (1.21-2.52)	2.31 (0.99-5.41)

Note. All analyses were based on weighted datasets. Odds ratios (ORs), Incidence rate ratios (IRRs) and 95% confidence intervals (CI) are presented in the table. The consistent engagement was the referenced group. Model 1 adjusted for predisposing and enabling factors (age, gender, educational level, race/ethnicity, marital status, employment/occupation, household wealth and life satisfaction). Model 2 adjusted for confounders in Model 1 and health-related covariates (depression, multimorbidity, ADLs, IADLs, and self-rated health status). Community activities (volunteering, charity work, educational courses, sports or social clubs, meetings of non-religious organisations), cognitive activities (reading, word games, cards, writing), home-based creative activities (gardening, baking, cooking, crafts, hobbies), and physical activities (exercise, walking).

© 2023 Gao Q et al. *JAMA Network Open.*
