# Supplementary material for: Associations of Social, Cultural, and Community Engagement With Health Care Utilization in the US Health and Retirement Study
Source: JAMA Netw Open. 2023 Apr 4;6(4):e236636. doi: 10.1001/jamanetworkopen.2023.6636 (PMC10074222; doi:10.1001/jamanetworkopen.2023.6636)
Supplement: Supplement 2. — Data Sharing Statement [file jamanetwopen-e236636-s002.pdf]

# Data Sharing Statement

Gao. Associations of Social, Cultural, and Community Engagement With Health Care Utilization in the US Health and Retirement Study. *JAMA Netw Open*. Published April 04, 2023. doi:10.1001/jamanetworkopen.2023.6636

**Data**
**Data available:** Yes
**Data types:** Deidentified participant data
**How to access data:** Raw data were generated at the RAND Center for the Study of Aging (https://hrsdata.isr.umich.edu/data-products/rand)
**When available:** With publication

**Supporting Documents**
**Document types:** None

**Additional Information**
**Who can access the data:** Data supporting the findings of this study are available from the author QG on request.
**Types of analyses:** NA
**Mechanisms of data availability:** The HRS data are obtained available at the RAND Center for the Study of Aging without investigator support.
